# Advanced Quantitative Lipoprotein Characteristics Do Not Relate to Healthy Dietary Patterns in Adults from a Mediterranean Area

**DOI:** 10.3390/nu13124369

**Published:** 2021-12-06

**Authors:** Marina Idalia Rojo-López, Esmeralda Castelblanco, Jordi Real, Marta Hernández, Mireia Falguera, Núria Amigó, Josep Julve, Núria Alonso, Josep Franch-Nadal, Minerva Granado-Casas, Dídac Mauricio

**Affiliations:** 1 Department of Endocrinology and Nutrition, Hospital de la Santa Creu i Sant Pau & Institut d’Investigació Biomèdica Sant Pau (IIB Sant Pau), 08041 Barcelona, Spain; nut.marina.rojo.l@gmail.com (M.I.R.-L.); jjulve@santpau.cat (J.J.); 2 Department of Internal Medicine, Endocrinology, Metabolism and Lipid Research Division, Washington University School of Medicine, St Louis, MO 63110, USA; esmeraldacas@gmail.com; 3 Center for Biomedical Research on Diabetes and Associated Metabolic Diseases (CIBERDEM), Instituto de Salud Carlos III, 28029 Madrid, Spain; jordi.real@gmail.com (J.R.); namigo@biosferteslab.com (N.A.); nalonso32416@yahoo.es (N.A.); josep.franch@gmail.com (J.F.-N.); 4 DAP-Cat Group, Unitat de Suport a la Recerca Barcelona, Institut Universitari d’Investigació en Atenció Primària Jordi Gol (IDIAP Jordi Gol), 08041 Barcelona, Spain; 5 Department of Endocrinology & Nutrition, University Hospital Arnau de Vilanova, 25198 Lleida, Spain; martahernandezg@gmail.com; 6 Lleida Institute for Biomedical Research Dr. Pifarré Foundation IRBLleida, University of Lleida, 25198 Lleida, Spain; mireiafalguera@hotmail.com; 7 Primary Health Care Centre Cervera, Gerència d’Atenció Primaria, Institut Català de la Salut, 25200 Lleida, Spain; 8 Department of Basic Medical Sciences, Universitat RoviraiVirgili, IISPV, 43007 Tarragona, Spain; 9 Biosfer Teslab, SL., 43204 Reus, Spain; 10 Department of Biochemistry and Molecular Biology, Universitat Autònoma de Barcelona, 08041 Barcelona, Spain; 11 Endocrinology and Nutrition Department, Hospital Universitari Germans Trias i Pujol, 08916 Badalona, Spain; 12 Department of Medicine, Universitat Autònoma de Barcelona, 08041 Barcelona, Spain; 13 Primary Health Care Centre Raval Sud, Gerència d’Atenció Primaria Barcelona, InstitutCatalà de la Salut, 08001 Barcelona, Spain; 14 Faculty of Medicine, University of Vic (UVIC/UCC), 08500 Vic, Spain

**Keywords:** Mediterranean diet, healthy eating, dietary pattern, advanced lipoprotein profile

## Abstract

We aimed to assess the potential relationship between dietary patterns (i.e., Mediterranean diet and healthy eating) and the advanced lipoprotein profile (ALP) in a representative cohort of the Mediterranean population. Thus, ALP data from 1142 participants, including 222 with type 1 (19.4%) and 252 type 2 diabetes (22.1%), and 668 subjects without diabetes were used to study cross-sectional associations between quantitative characteristics of lipoproteins and adherence to the Mediterranean diet. The alternate Mediterranean diet score (aMED) and the alternate healthy eating index (aHEI) were calculated. The ALP was determined by nuclear magnetic resonance (NMR) spectrometry. Bivariable and multivariable analyses were performed. Participants in the third tertile of the aMED showed higher levels of low-density lipoprotein triglycerides (LDL-TG) (mean (SD) 17.5 (5.0); *p* = 0.037), large high-density lipoprotein particles (HDL-P) (0.3 (0.1); *p* = 0.037), and medium low-density lipoprotein particles (LDL-P) (434.0 (143.0); *p* = 0.037). In comparison with participants in the second and first tertiles of the aHEI, participants in the third tertile had higher levels of LDL-TG (17.7 (5.0); *p* = 0.010), and large HDL-P (0.3 (0.1); *p* = 0.002), IDL-C (11.8 (5.0); *p* = 0.001), intermediate-density lipoprotein triglycerides (IDL-TG) (13.2 (4.2); *p* < 0.001), LDL-TG (17.7(5.0); *p* = 0.010), high-density lipoprotein triglycerides (HDL-TG) (14.5 (4.4); *p* = 0.029,) large HDL-P (0.3 (0.1); *p* = 0.002) and very–low-density lipoprotein particles (VLDL-P) size (42.1 (0.2); *p* = 0.011). The adjusted-multivariable analysis for potential confounding variables did not show any association between the lipoproteins and dietary patterns (i.e., aMED and aHEI). In conclusion, none of the quantitative characteristics of lipoproteins were concomitantly associated with the extent of adherence to the Mediterranean diet measured using the aMED or aHEI scores in the studied population. Our findings also revealed that people with the highest adherence were older, had a higher body mass index (BMI) and more frequently had dyslipidemia, hypertension, or diabetes than those with the lowest adherence to the Mediterranean diet (MDiet). Thus, further research may be needed to assess the potential role of the dietary pattern on the ALP.

## 1. Introduction

Total cholesterol, triglycerides, high-density lipoprotein (HDL), and low-density lipoprotein (LDL) determined by conventional lipid analysis are commonly used to identify subjects with metabolic diseases at high cardiovascular (CV) risk [1,2]. However, the basic determination of such serum lipid concentrations may not necessarily reflect potentially informative changes in other quantitative characteristics of lipoproteins (i.e., size, and lipoprotein subclasses that may remain hidden). In this context, the advanced lipoprotein profile (ALP) analysis by nuclear magnetic resonance (NMR) spectrometry [3] provides the concentration of the major classes of lipoprotein particles (i.e., very-low-density lipoprotein [VLDL], LDL, and HDL), and the number and size of the different subclasses [2]. Of note, LDL-particle (LDL-P) and HDL-particle (HDL-P) levels are more sensitive for predicting CV risk in comparison with classical lipid profiles currently assessed in clinical practice [4]. In this regard, elevated serum concentrations of smaller LDL-P and HDL-P appear to be more atherogenic than other particles [5]. Consistently, the Mediterranean Diet (MDiet), which is rich in “healthy fats”, has been associated with lower serum LDL concentrations and hence a reduced risk of CV disease [6].

The potential relationship between dietary patterns and ALP is controversial [7,8,9,10,11,12,13,14,15]. On the one hand, a reduction of total LDL-P number, intermediate-density lipoprotein (IDL), medium and small-LDL concentration, and elevated concentrations of larger LDL was shown in subjects fed with a MDiet supplemented with nuts in a post-hoc, large-scale study (PREDIMED) [7]. Additionally, consumption of the traditional MDiet enriched with virgin olive oil also increased LDL-P size and large HDL concentration [7,8]. Data from a post-hoc analysis showed normalization of the HDL-P size with a hypocaloric modified Dietary Approaches to Stop Hypertension (DASH) diet and exercise intervention in subjects with metabolic syndrome [11]. Moreover, a post-hoc analysis from the Finnish Gestational Diabetes Prevention Study (RADIEL) trial showed that a healthy dietary pattern was associated with a low concentration of HDL-P, as well as a reduced VLDL-P size [12]. In addition, a recent study performed with a large cohort of healthy women (*n* = 26,034) showed that dietary omega-3 fatty supplementation increased HDL size and large HDL-particles [13]. Furthermore, a prospective study performed with a large cohort of healthy women observed an association between the MDiet and reduced cardiovascular risk through HDL and VLDL measures [14]. These authors also described a weak association with LDL size and particles, but not LDL or total cholesterol [14]. Finally, a cross-sectional study performed with middle-to older-aged adults found that a healthy dietary pattern was associated with a lower concentration of large and medium VLDL and total and small LDL-P [15]. In contrast, other interventional studies performed with the MDiet did not find changes in the ALP in healthy women [9,10].

To our knowledge, this is the first study specifically assessing the potential relationship between adherence to Mediterranean dietary patterns and the advanced quantitative characteristics of lipoproteins. We hypothesized that people with the highest score to the MDiet and aHEI would be associated with a healthier ALP. Thus, the aim of the study was to assess the relationship between the dietary pattern and ALP characteristics in adults with and without diabetes mellitus from a Mediterranean region.

## 2. Materials and Methods

### 2.1. Study Design

This was a cross-sectional study that included 1142 participants from three different cohorts from Catalonia (Spain). The participants were recruited from two geographical areas in northeastern Spain (Lleida and Barcelona). The study population included 222 individuals diagnosed with type 1 diabetes mellitus (T1DM), 252 subjects with type 2 diabetes mellitus (T2DM) and 668 individuals without diabetes. Detailed descriptions of the study cohorts can be found in previously published studies [16,17,18]. The inclusion criteria of individuals with T1DM were as follows: diagnosis of T1DM of more than 1-year duration and age > 18 years. In the T2DM group, individuals with a diagnosis of T2DM aged between 40 and 75 years were included. In the group without diabetes, the inclusion criteria were being over 25 years old and having normal glucose tolerance. The exclusion criteria for all participants were as follows: being a healthcare professional, participants who showed mental disorders, a history of previous cardiovascular disease or diabetic foot disease, chronic kidney disease (defined as an estimated glomerular filtration rate < 60 mL/min or a urine albumin/creatinine ratio over 300 mcg/g), pregnancy, and other conditions that could influence the study results. Furthermore, individuals without diabetes were also excluded if they had a fasting glucose and glycated hemoglobin (HbA1c) values above 100 mg/dL and 5.7% (39 mmol/mol), respectively. The local Ethics Committee from the different participating institutions, Primary Health Care University Research Institute (IDIAP) Jordi Gol (P12/043), University Hospital Arnau de Vilanova (CEIC 1079), and University Hospital Germans Trias i Pujol (PI-15-147) approved the study. Written informed consent was obtained from all the participants.

### 2.2. Clinical Data

Blood samples were collected to determine biochemical variables using standard lab procedures. Furthermore, clinical and sociodemographic data were collected from anamnesis and medical records. Anthropometric variables (i.e., weight, height, and body mass index [BMI]) were obtained by standardized methods [19,20]. The use of any antihypertensive or lipid-lowering drugs was used to define the presence of hypertension and dyslipidemia, respectively. Regular physical activity was defined as performing any kind of physical activity (such as walking) for more than 25 min/day (i.e., 4 Metabolic Equivalents [METS]), and sedentarism was defined if the physical activity lasted less than 25 min/day according to Bernstein et al. [21] and Cabrera de León et al. [22]. Tobacco exposure was determined including former and current smokers.

### 2.3. Dietary Pattern Assessment

Diet was assessed using the validated 101-item semiquantitative food frequency questionnaire consumption (FFQC) [23,24]. This was individually administered by specialized and trained researchers. The FFQC collects data on habitual food consumption in the past year prior to the visit. The dietary pattern was assessed using two dietary quality indexes: the alternate Mediterranean Diet score (aMED) and the alternate Healthy Eating Index (aHEI) [25,26]. The aMED scores range from 0 (minimum) to 9 (maximum). This index includes essential components of the traditional MDiet such as healthy fats derived from olive oil, nuts, and fish, a low intake of saturated fat, and a diet rich in legumes, vegetables, fruits, cereals, meat, and wine [25]. The aHEI scores range from 0 (unhealthy eating) to 80 (healthy eating). The evaluation includes the consumption of vegetables, fruit, nuts and soy, white and red meat, cereal fiber, polyunsaturated-to-saturated fatty acid ratio, trans fat, and alcohol consumption [26]. Three groups (tertiles) (i.e., low, moderate, and high adherence to the MDiet diet) were defined as an aMED score of 0–3, 4 and 5–9, respectively, and an aHEI score of 20–37, 38–43, and 44–68 (representing the first [T1], second [T2] and third tertile [T3]).

The US Department of Agriculture food composition tables and other Spanish and English food sources were used to estimate nutrient intake [27,28,29]; this was adjusted for energy intake.

### 2.4. Advanced Lipoprotein Profile

The advanced lipoprotein profile of plasma samples was performed using two-dimensional diffusion-ordered 1H-NMR spectroscopy (2D DOSY) Liposcale^®^ [4]. The Liposcale^®^ test consists of the analysis of the attenuation of the signals of lipid peaks when they are subjected to a known magnetic field gradient. Under the influence of radiofrequency pulses, lipoproteins resonate at slightly different frequencies depending on their size, generating a spectrum of these frequencies; thus, the smaller the lipoprotein, the lower the resonance frequency of the lipids in its nucleus. Therefore, using NMR-based analytical techniques, it is possible to measure the size of lipoproteins directly [4,30]. Nine lipoprotein subclasses (large, medium, and small VLDL, LDL, and HDL), the size and particle number of three different classes of lipoproteins (VLDL, LDL, and HDL) and the lipid concentrations (i.e., triglycerides and cholesterol) were assessed using this method.

### 2.5. Statistical Analyses

A descriptive analysis of all the variables was performed, summarizing the quantitative variables with the mean and standard deviation and the qualitative variables with the frequency and the percentage. A comparison of the characteristics between groups (i.e., low, moderate, and high adherence to the MDiet according to the T1, T2 and T3 tertiles of the aMED and aHEI) was carried out using the chi-square test for qualitative variables, and the ANOVA test, for quantitative variables. The compare Groups R package [31] was used to perform the calculations and tables.

To assess the advanced lipoprotein profile and the dietary quality index of the participants, each lipoprotein parameter was compared between the groups using the ANOVA test. Furthermore, adjusted analysis of association of each lipoprotein versus aMED and aHEI using regression linear models.

The variables included in the models was the known confounding factors related to lipoprotein profile and the dietary quality: diabetes mellitus, sex, age, physical activity, hypertension, dyslipidemia, BMI, and tobacco exposure. The family type I error was prefixed at 0.05, but the Benjamini and Hochberg corrections were applied for multiple comparisons to control the false discovery rate (p. adjust function from stats R package). The statistical analyses were performed using R3.6.1 [32].

## 3. Results

The clinical and demographic characteristics of the participants related to the dietary quality indexes are shown in Table 1. Participants with a greater adherence to the aMED tended to be older, while participants with a greater adherence to the aHEI were also older and tended to be women (64%). Participants in the second tertile of the aMED and the third tertile of the aHEI (moderate and high adherence, respectively) showed poorer glycemic control (mean (SD) 6.7 (1.5); *p* < 0.001, and 6.0 (1.2); *p* < 0.001, respectively), a higher BMI (28.0 (5.5); *p* = 0.047, and 27.9 (5.1); *p* < 0.001, respectively) and higher systolic blood pressure (SBP) (128.0 (20.0); *p* = 0.044, and 130.0 (18.6); *p* < 0.001, respectively) in comparison with participants in the first and third tertiles of the aMED and participants in the first and second tertiles of the aHEI. Moreover, participants in the second tertile of the aMED and aHEI showed a higher waist circumference (97.3 (14.3); *p* = 0.013, and 96.0 (13.9); *p* = 0.054, respectively) compared with the first and third tertiles of the aMED and aHEI. Additionally, similar results were found when stratifying the study sample according to the type of diabetes (Tables S1 and S2).

Data are shown as *n* (%) for categorical variables and mean (SD) for continuous variables. BMI, body mass index; DBP, diastolic blood pressure; HbA1c, glycated hemoglobin; HDL-cholesterol, high density lipoprotein-cholesterol; LDL-cholesterol, low density lipoprotein-cholesterol; SBP, systolic blood pressure. *p* was calculated using ANOVA and Chi-square to compare groups. T1, poor adherence; T2, moderate adherence; and T3, high adherence.

### 3.1. Dietary Intake of the Study Subjects

Furthermore, as expected, participants with higher scores of aMED and aHEI showed a higher nutrient intake of polyunsaturated fat, omega 3 and omega 6 fatty acids in comparison with participants allocated in the first and second tertiles (Table S3). Moreover, participants allocated in the third tertile of aMED and aHEI showed a healthier daily food intake, with exception for the consumption of sugar, in comparison with participants in the first and second tertiles (Table S3).

### 3.2. Advanced Lipoprotein Profile and Mediterranean Diet

The ALP according to the dietary quality index groups is shown in Table 2. Participants in the third tertile of the aMED (high adherence) showed higher levels of LDL-TG (mean (SD) 17.5 (5.0); *p* = 0.037), medium LDL-P (434.0 (143.0); *p* = 0.037), and large HDL-P (0.3 (0.1); *p* = 0.037) compared with participants in the first and second tertiles. Furthermore, participants in the second tertile of the aMED showed higher levels of IDL-C (11.3 (5.1); *p* = 0.037) compared with participants in the first and third tertiles. In terms of the ALP and aMED, no differences were observed between T1DM and T2DM participants (Table S5). However, control participants in the third tertile of the aMED showed lower levels of total, large and small VLDL-P number (mean (SD) 41.4 (24.7); *p* = 0.021, 1.0 (0.5); *p* = 0.012, and, 36.3 (22.3); *p* = 0.021, respectively), VLDL-C (10.0 (7.7); *p* = 0.039) and VLDL-TG (59.4 (33.9); *p* = 0.021), in comparison with control participants in the second and third tertiles (Table S4). Additional analysis of the study subjects by age revealed that the ALP in participants under age 50 with a higher adherence to the MDiet showed higher levels of LDL particles, LDL-C, LDL-TG, IDL-C, large HDL, and non-HDL particles in comparison with those in the first and second tertiles of the aMED (Table S6). Further, taking the same approach in men did not reveal any differences (Table S7). However, women under age 50 with a higher adherence to the MDiet showed higher levels of LDL particles, LDL-C, LDL-TG, IDL-C, large HDL, and non-HDL particles in comparison with those women in the first and second tertiles of the aMED (Table S8). The multivariable analysis between lipoprotein subclasses and the aMED score did not show any association when adjusting for potential confounding variables (Table 3).

Data are shown as mean (SD). HDL, high-density lipoprotein; HDL-C, cholesterol content in HDL; HDL-P, HDL particles; HDL-TG, triglyceride content in HDL; IDL, intermediate-density lipoprotein; IDL-C, cholesterol content in IDL; IDL-TG, triglyceride content in IDL; LDL, low-density lipoprotein; LDL-C, cholesterol content in LDL; LDL-P, LDL particles; LDL-TG, triglyceride content in LDL; NMR, nuclear magnetic resonance; VLDL, very low density lipoprotein; VLDL-C, cholesterol content in VLDL; VLDL-P, VLDL particles; VLDL-TG, triglyceride content in VLD. T1, poor adherence; T2, moderate adherence; and T3, high adherence.

Each coefficient estimated ± standard error was computed by multiple linear regression models adjusted by: diabetes mellitus, sex, age (years), physical activity, hypertension, dyslipidemia, body mass index (Kg/m^2^), tobacco exposure. *p*: *p* value was corrected by Benjamini and Hochberg for multiple comparisons.

### 3.3. Advanced Lipoprotein Profile and Healthy Eating Pattern

Participants in the third tertile of the aHEI showed higher levels of IDL-C (mean (SD) 11.8 (5.0); *p* = 0.001), IDL-TG (13.2 (4.2); *p* ≤ 0.001), LDL-TG (17.7 (5.0); *p* = 0.010), HDL-TG (14.5 (4.4); *p* = 0.029) and large HDL-P (0.3 (0.1); *p* = 0.002) (Table 2) compared with participants in the first and second tertiles. However, participants in the third tertile of the aHEI showed lower levels of VLDL-P size (mean (SD) 42.1(0.2); *p* = 0.011) in comparison to participants in the first and second tertiles. No differences were observed in terms of the ALP and the aHEI in participants with T2DM (Table S5). However, participants with T1DM in the third tertile of the aHEI showed higher levels of total, large and medium HDL-P (mean (SD) 31.0 (5.4); *p* = 0.046, 0.30 (0.04); *p* = 0.033 and, 11.5 (2.0); *p* = 0.046, respectively), compared with T1DM participants in the first and second tertiles (Table S5). In addition, control participants in the third tertile of the aHEI showed higher levels of large and medium LDL-P (mean (SD) 197.0 (39.3); *p* = 0.036, and 464.0 (148.0); *p* = 0.036, respectively), large and medium HDL-P (0.29 (0.0); *p* = 0.018, and 9.8 (2.1); *p* = 0.091, respectively), IDL-TG (17.7 (5.4); *p* = 0.008) and non-HDL-P (1388.0 (301.0); *p* = 0.022), in comparison with control participants in the first and second tertiles (Table S5). However, no differences were observed between the ALP and the aHEI in participants stratified by age and sex groups (Tables S6–S8). The multivariable analysis between lipoprotein subclasses and the aHEI score did not show any association after adjusting for potential confounding variables (Table 3).

## 4. Discussion

The present study assessed the characteristics of circulating lipoproteins and their potential relationship with dietary patterns (i.e., aMED and aHEI) in a large cohort of Catalan adult people recruited from the northeastern region of Spain. Despite participants with higher aMED and aHEI scores showing a higher concentration of LDL-TG and large HDL-P, no association was found between the ALP and the dietary pattern (i.e., aMED and aHEI) after adjusting for confounding variables. However, participants with the highest adherence to the MDiet and higher scores of aHEI were older, had a higher BMI and more frequently had dyslipidemia, hypertension, and diabetes versus those with the lowest adherence. The findings that participants with more CV-related morbidities have higher aMED and aHEI scores may be due to them adhering to educational dietary interventions with the aim of improving their CV health.

A previous study showed that higher intake of either red or white meat, was associated with higher concentrations of LDL-C and large LDL particles [33]. Furthermore, higher intake of fish in postmenopausal women has been associated with higher levels of LDL-C, while dietary omega-3 was associated with increased HDL size and large HDL particles [13]. Moreover, fish and seafood consumption has been associated with higher levels of large LDL-C and HDL-C particles [34]. In the present study, participants with higher aMED and aHEI scores had a higher consumption of white meat, fish and seafood, and lower intake of red meat and processed meat. However, they had an ALP profile associated with higher CV risk, which is in contrast with the published studies [13,34,35].

In the context of the MDiet, the concept that an elevated adherence to this type of diet relates to decreased LDL atherogenicity has been reported in some [7,8], but not all studies [9,10]. Similarly, the serum concentration of LDL-P did not differ among the different subgroups of either the aMED or aHEI. Higher scores of health eating index has been inversely correlated with TG, large VLDL-P, medium VLDL-P, IDL, small LDL-P, small HDL-P and VLDL size and positively correlated with large LDL-P, large HDL and both LDL and HDL size [36]. In contrast, total cholesterol, LDL-C, TG, and HDL-C concentrations did not differ among different tertiles of adherence to the aHEI. i.e., low, moderate and high adherence. Importantly, our ALP analysis uncovered hidden proatherogenic lipoprotein characteristics in participants with the highest aMED and aHEI scores. Indeed, the serum concentration of medium-sized LDL was significantly increased in people with the highest adherence according to the aMED. Since the medium LDL subclass determined using the Liposcale^®^ method in our subjects corresponds in size to the smaller LDL according to the Liposcience^®^ range criteria, our findings suggest that smaller and hence more atherogenic LDLs are present in the circulation in the group of participants with the highest adherence to the MDiet. In line with this view, medium LDLs have been associated with an elevated incidence of CV disease [37], by virtue of their elevated susceptibility to be oxidized [8].

Compelling evidence suggests that higher levels of HDL size, and HDL-P concentration are inversely associated with CV risk [38]. Furthermore, elevated large HDL are considered antiatherogenic particles [7,15,35]. Although in a study the subjects with the highest adherence scores according to the aHEI showed an improved conventional lipid profile and lower risk of CV disease [39], this was not reproduced in a recent independent study where the adherence to healthy dietary recommendations was not associated with a lower CV disease risk [40]. Our data show that the serum concentration of the large HDL-P subclass was significantly elevated in participants with higher score of aMED and aHEI. Although the actual clinical significance of this finding needs further investigation and could be irrelevant, as suggested by our study and others.

Although previous studies have described that women have an improved ALP in comparison with men [41], we did not observe significant differences in the ALP between men and women. Nevertheless, post-menopausal women have been reported to have an atherogenic lipid profile due to an elevation of almost all cholesterol-carrying particles [42]. However, in the current study, only younger women (aged below 50) with a higher adherence to the MDiet showed higher levels of LDL particles, LDL-C, LDL-TG, IDL-C, large HDL and non-HDL particles. We should be cautious about the interpretation of these findings as this is just a sub-analysis of our sample that should not lead to any conclusion. Moreover, in contrast with our findings, an altered lipoprotein profile has been reported in subjects with diabetes compared with normoglycemic participants [43].

The current study had several limitations. A causal relationship between study variables cannot be established due to the cross-sectional study design. Additionally, the impact of possible changes in dietary habits produced over time could not be addressed in this study. In addition, the absence of differences in the ALP between dietary patterns may be, at least in part, explained by the sample size. Furthermore, the low variability of the dietary patterns may be partially attributed to the fact that all participants belong to the same region. Moreover, this study does not account for genetic variations potentially contributing to particle size. Nevertheless, the study has several strengths. This is the first study to assess the relationship between the ALP and the dietary pattern measured by the aMED and aHEI in adults from a Mediterranean area. Since the fatty acid intake profile has an impact on ALP, a comprehensive assessment of their intake was better assessed by the two dietary indexes. These dietary quality indexes allowed us to identify, on the one hand, trans-fat intake and the ratio of polyunsaturated to saturated fats through the aHEI and, on the other hand, the ratio of monounsaturated lipids to saturated lipids through aMED [25,26]. The large number of participants and the multicenter design may allow the results to be translated to other populations. Of note, we included a well-characterized sample without the presence of previous CV disease, while still maintaining the variability of participant characteristics and lifestyles that are a benefit of real-world studies. Finally, we used the NMR spectroscopy to analyze the ALP, which is considered the most precise method to assess lipoprotein variations.

## 5. Conclusions

The quantitative characteristics of lipoproteins revealed by the ALP analysis were not associated with any of the dietary patterns in the studied population after adjusting for confounding variables. However, the potential role of the dietary pattern on the ALP needs to be further studied.

## Figures and Tables

**Table 1:** Clinical characteristics according to adherence to the diet assessed using dietary quality indexes.

Characteristics	aMED				aHEI			
	T1 (0–4)	T2 (4)	T3 (5–9)	*p*	T1 (20–38)	T2 (38–44)	T3 (44–68)	*p*
	(*N* = 540)	(*N* = 234)	(*N* = 368)		(*N* = 426)	(*N* = 369)	(*N* = 347)	
Age (years)	48.3 (12.7)	51.6 (13.0)	52.7 (12.1)	<0.001	47.0 (12.2)	49.7 (12.4)	55.3 (12.2)	<0.001
Sex (women)	291 (53.9)	122 (52.1)	218 (59.2)	0.158	206 (48.4)	203 (55.0)	222 (64.0)	<0.001
Educational level				0.238				0.323
Undergraduate	453 (84.8)	187 (82.0)	294 (80.6)		204 (81.9)	363 (81.5)	295 (85.5)	
Graduate or higher	81 (15.2)	41 (18.0)	71 (19.5)		76 (18.1)	67 (18.5)	50 (14.5)	
Tobacco exposure	294 (54.5)	114 (48.7)	188 (51.1)	0.001	263 (61.7)	184 (49.9)	149 (42.9)	<0.001
Regular physical activity	326 (61.0)	144 (63.2)	259 (70.8)	0.010	254 (61.1)	249 (67.8)	226 (65.5)	0.129
BMI (kg/m2)	27.0 (5.2)	28.0 (5.5)	27.1 (5.0)	0.047	26.5 (4.8)	27.6 (5.6)	27.9 (5.1)	<0.001
Waist (cm)	94.9 (13.3)	97.3 (14.3)	94.0 (13.2)	0.013	93.8 (13.3)	96.0 (13.9)	95.7 (13.2)	0.054
SBP (mmHg)	126.0 (17.7)	128.0 (20.0)	128.0 (18.1)	0.044	124.0 (17.2)	127.0 (19.0)	130.0 (18.6)	<0.001
DBP (mmHg)	76.5 (9.7)	76.1 (10.4)	75.8 (10.1)	<0.001	76.2 (9.2)	77.2 (10.2)	75.3 (10.6)	0.043
Hypertension	112 (20.7)	72 (30.8)	111 (30.2)	0.001	71 (16.7)	95 (25.7)	129 (37.2)	<0.001
Dyslipidemia	133 (24.6)	89 (38.0)	126 (34.2)	<0.001	96 (22.5)	106 (28.7)	146 (42.1)	<0.001
HbA1c (mmol/mol)	43.8 (14.3)	49.3 (16.1)	48.6 (15.0)	<0.001	42.5 (13.1)	46.2 (14.3)	51.6 (16.7)	<0.001
HbA1c (%)	6.2 (1.3)	6.7 (1.5)	6.6 (1.4)	<0.001	6.0 (1.2)	6.4 (1.3)	6.9 (1.5)	<0.001
Total cholesterol (mg/dL)	192.0 (36.1)	191.0 (35.8)	194.0 (36.8)	0.657	193.0 (35.6)	190.0 (36.2)	194.0 (37.0)	0.336
HDL-cholesterol (mg/dL)	58.0 (16.4)	57.6 (15.2)	59.6 (15.2)	0.214	58.8 (16.1)	58.5 (16.4)	57.9 (14.9)	0.724
LDL-cholesterol (mg/dL)	114.0 (30.0)	112.0 (31.1)	116.0 (31.8)	0.419	114.0 (30.0)	113.0 (31.2)	116.0 (31.4)	0.506
Triglycerides (mg/dL)	108.0 (97.0)	111.0 (73.8)	101.0 (58.8)	0.295	107.0 (104)	102.0 (68.7)	110.0 (61.9)	0.477

High-density lipoprotein (HDL). Low-density lipoprotein (LDL).

**Table 2:** Nuclear magnetic resonance -assessed advanced lipoprotein profile according to adherence to the diet assessed using dietary quality indexes.

NMR Variable	aMED				aHEI			
	T1 (0–4)	T2 (4)	T3 (5–9)	*p*	T1 (20–38)	T2 (38–44)	T3 (44–68)	*p*
	(*N* = 540)	(*N* = 234)	(*N* = 368)		*(N* = 426)	(*N* = 369)	(*N* = 347)	
VLDL-P number (nmol/L)								
Total	49.7 (41.4)	52.2 (41.2)	45.2 (30.7)	0.189	48.1 (42.3)	47.4 (36.5)	51.0 (34.8)	0.454
Large	1.2 (0.9)	1.3 (0.8)	1.1 (0.6)	0.113	1.2 (0.9)	1.2 (0.8)	1.2 (0.7)	0.752
Medium	4.9 (6.0)	5.0 (5.6)	4.4 (3.3)	0.192	4.9 (6.7)	4.7 (4.7)	4.8 (3.4)	0.824
Small	43.5 (35.2)	45.9 (35.7)	39.7 (27.3)	0.189	42.0 (35.5)	41.6 (31.8)	45.0 (31.1)	0.385
VLDL-P composition (mg/dL)								
VLDL-C	12.2 (11.7)	12.7 (11.4)	11.1 (9.71)	0.236	11.7 (11.7)	11.5 (10.7)	12.7 (10.6)	0.431
VLDL-TG	71.7 (63.2)	75.0 (61.6)	64.7 (42.8)	0.189	69.6 (66.2)	68.2 (53.6)	72.7 (48.1)	0.613
VLDL-P size (nm)	42.1 (0.2)	42.1 (0.2)	42.1 (0.2)	0.424	42.1 (0.2)	42.1 (0.2)	42.1 (0.2)	0.011
LDL-P number (nmol/L)								
Total	1302.0 (257.0)	1324.0 (264.0)	1343.0 (273.0)	0.086	1308.0 (264.0)	1311.0 (262.0)	1343.0 (266.0)	0.196
Large	183.0 (33.5)	182.0 (36.8)	187.0 (36.5)	0.189	184.0 (34.8)	182.0 (34.5)	186.0 (36.3)	0.613
Medium	408.0 (130.0)	416.0 (138.0)	434.0 (143.0)	0.037	415.0 (135.0)	412.0 (134.0)	428.0 (140.0)	0.385
Small	710.0 (138.0)	725.0 (133.0)	722.0 (136.0)	0.236	708.0 (141.0)	717.0 (134.0)	728.0 (134.0)	0.149
LDL-P composition (mg/dL)								
LDL-C	127.0 (25.5)	128.0 (26.6)	131.0 (27.8)	0.101	128.0 (26.0)	127.0 (26.6)	130.0 (27.0)	0.385
LDL-TG	16.4 (5.0)	17.0 (5.2)	17.5 (5.0)	0.037	16.5 (5.2)	16.6 (4.9)	17.7 (5.0)	0.010
LDL-P size (nm)	21.0 (0.3)	21.0 (0.3)	21.0 (0.3)	0.554	21.0 (0.3)	21.0 (0.3)	21.0 (0.3)	0.385
HDL-P number (μmol/L)								
Total	29.0 (6.0)	29.5 (6.1)	29.9 (5.5)	0.101	29.2 (6.0)	29.2 (6.0)	29.9 (6.0)	0.378
Large	0.3 (0.1)	0.3 (0.1)	0.3 (0.1)	0.037	0.3 (0.1)	0.3 (0.1)	0.3 (0.1)	0.002
Medium	9.4 (2.3)	9.4 (2.4)	9.6 (2.2)	0.189	9.4 (2.3)	9.5 (2.4)	9.6 (2.2)	0.385
Small	19.4 (4.4)	19.9 (4.4)	20.0 (4.0)	0.101	19.6 (4.4)	19.5 (4.2)	20.0 (4.1)	0.385
HDL-P composition (mg/dL)								
HDL-C	56.1 (13.6)	56.6 (13.8)	57.9 (12.5)	0.113	56.5 (13.1)	56.7 (13.9)	57.3 (12.9)	0.564
HDL-TG	13.7 (4.7)	14.0 (4.3)	14.0 (4.6)	0.355	13.5 (4.7)	13.6 (4.5)	14.5 (4.4)	0.029
HDL-P size (nm)	8.2 (0.1)	8.2 (0.1)	8.2 (0.1)	0.688	8.2 (0.1)	8.2 (0.1)	8.2 (0.1)	0.860
IDL-P composition (mg/dL)								
IDL-C	10.3 (4.8)	11.3 (5.1)	11.2 (4.7)	0.037	10.3 (4.8)	10.5 (4.7)	11.8 (5.0)	0.001
IDL-TG	12.0 (3.9)	12.8 (4.2)	12.5 (3.9)	0.086	11.9 (3.7)	12.0 (3.9)	13.2 (4.2)	<0.001
Other atherogenic variables								
Non-HDL-P (nmol/L)	1322.0 (265.0)	1346.0 (269.0)	1359.0 (278.0)	0.113	1326.0 (272.0)	1330.0 (267.0)	1364.0 (272.0)	0.190
Total-P/HDL-P	48.5 (14.0)	48.8 (14.8)	48.2 (14.2)	0.784	48.3 (13.9)	48.6 (14.5)	48.6 (14.3)	0.824
LDL-P/HDL-P	46.7 (13.3)	46.8 (13.9)	46.6 (13.6)	0.904	46.6 (13.2)	46.8 (13.8)	46.8 (13.6)	0.860

Very–low-density lipoprotein particles (VLDL-P). High-density lipoprotein particles (HDL-P). High-density lipoprotein triglycerides (HDL-TG).

**Table 3:** Adjusted associations between each advanced lipoprotein profile and the alternate Mediterranean Diet (aMED) and alternate healthy eating index (aHEI) scores. Coefficients were estimated by multiple linear regression models.

NMR Variable	aMED		aHEI	
	Coefficient	*p*	Coefficient	*p*
VLDL-P number (nmol/L)				
Total	−1.096 (0.620)	0.407	−0.254 (0.158)	0.172
Large	−0.027 (0.013)	0.407	−0.007 (0.003)	0.142
Medium	−0.156 (0.091)	0.407	−0.035 (0.023)	0.172
Small	−0.913 (0.531)	0.407	−0.213 (0.136)	0.172
LDL-P number (nmol/L)				
Total	9.845 (4.645)	0.611	1.010 (1.189)	0.142
Large	0.807 (0.618)	0.611	0.166 (0.158)	0.287
Medium	5.503 (2.389)	0.611	0.558 (0.612)	0.142
Small	3.535 (2.288)	0.683	0.286 (0.585)	0.210
HDL-P number (μmol/L)				
Total	0.091 (0.095)	0.625	−0.016 (0.024)	0.407
Large	0.001 (0.001)	0.625	0.000 (0.000)	0.452
Medium	0.013 (0.034)	0.974	0.000 (0.009)	0.703
Small	0.077 (0.073)	0.611	−0.015 (0.019)	0.389

## Data Availability

The data presented in this study are available on request from the corresponding author.

## Supplementary Materials

The following are available online at https://www.mdpi.com/article/10.3390/nu13124369/s1, Table S1: Clinical characteristics and the alternate Mediterranean Diet score (aMED) by population, Table S2: Clinical characteristics and the alternate Healthy Eating Index (aHEI) by population, Table S3: Daily nutrient intake by the alternate Mediterranean Diet score and the alternate Healthy Eating Index, Table S4: The advanced lipoprotein profile and the alternate Mediterranean Diet score (aMED) by population, Table S5: The advanced lipoprotein profile and the alternate Healthy Eating Index (aHEI) scores stratified by study group, Table S6: The advanced lipoprotein profile, the alternate Mediterranean Diet score and the alternate Healthy Eating Index by age groups, Table S7: The advanced lipoprotein profile, the alternate Mediterranean Diet score and the alternate Healthy Eating Index by age groups in men, Table S8: The advanced lipoprotein profile, the alternate Mediterranean Diet score and the alternate Healthy Eating Index by age groups in women.
