# Supplementary material for: Advanced Quantitative Lipoprotein Characteristics Do Not Relate to Healthy Dietary Patterns in Adults from a Mediterranean Area
Source: Nutrients. 2021 Dec 6;13(12):4369. doi: 10.3390/nu13124369 (PMC8706087; doi:10.3390/nu13124369)
Supplement: Supplementary file 1 [file nutrients-13-04369-s001.zip › nutrients-1458909-supplementary.pdf]

Supplementary Materials

Table S1: Clinical characteristics and the alternate Mediterranean Diet score (aMED) by population

Characteristics	Controls				T1DM				T2DM			
	T1 [0,4) (N= 371)	T2 [4] (N= 110)	T3 [5,9] (N=187)	p	T1 [0,4) (N= 90)	T2 [4] (N= 54)	T3 [5,9] (N=78)	p	T1 [0,4) (N= 79)	T2 [4] (N= 70)	T3 [5,9] (N=103)	p
Age (years)	48.0 (13.1)	50.0 (13.4)	51.3 (12.2)	0.014	42.9 (10.4)	44.4 (11.7)	46.4 (9.9)	0.098	56.1 (9.5)	59.5 (8.6)	59.9 (9.8)	0.017
Sex (women)	202 (54.4)	65 (59.1)	124 (66.3)		49 (54.4)	25 (46.3)	45 (57.7)		40 (50.6)	32 (45.7)	49 (47.6)	
Educational level				0.444				0.159				0.859
Undergraduate	311 (84.0)	87 (80.6)	149 (80.2)		68 (80.0)	35 (70.0)	51 (67.1)		69 (93.2)	63 (92.6)	92 (91.1)	
Graduate or higher	59 (15.9)	21 (19.4)	37 (19.9)		17 (20.0)	15 (30.0)	25 (32.9)		5 (6.8)	5 (7.3)	9 (8.9)	
Tobacco exposure	203 (54.7)	55 (50)	90 (48.1)	0.055	45 (49.4)	26 (48.2)	41 (52.6)	0.240	46 (58.2)	33 (47.1)	57 (55.3)	0.270
Regular physical activity	230 (62.8)	76 (73.1)	142 (76.8)	0.002	59 (66.3)	38 (70.4)	64 (82.1)	0.066	37 (46.8)	30 (42.9)	53 (51.5)	0.532
BMI (Kg/m2)	26.2 (4.6)	26.3 (4.7)	25.8 (4.2)	0.593	25.8 (4.6)	25.5 (3.4)	25.5 (3.7)	0.858	32.0 (5.5)	32.5 (5.3)	30.8 (5.5)	0.084
Waist (cm)	93.7 (12.4)	94.6 (13.3)	91.8 (11.7)	0.114	89.5 (12.6)	88.5 (11.9)	87.8 (11.8)	0.678	107 (11.4)	108 (10.6)	103 (12.3)	0.012
SBP (mmHg)	123.0 (15.9)	122.0 (19.3)	123.0 (15.7)	0.744	127.0 (17.5)	127.0 (18.0)	126.0 (17.6)	0.887	138.0 (20.3)	140.0 (17.4)	139.0 (18.3)	0.685
DBP (mmHg)	76.8 (9.65)	76.6 (10.6)	77.0 (9.62)	0.934	74.8 (9.19)	74.9 (9.71)	72.7 (10.0)	0.283	77.1 (10.2)	76.3 (10.7)	76.1 (10.6)	0.806
Hypertension	49 (13.2)	15 (13.6)	35 (18.7)	0.209	19 (21.1)	18 (33.3)	18 (23.1)	0.236	44 (55.7)	39 (55.7)	58 (56.3)	0.995
Dyslipidemia	62 (16.7)	27 (24.5)	48 (25.7)	0.024	38 (42.2)	23 (42.6)	30 (38.5)	0.852	33 (41.8)	39 (55.7)	48 (46.6)	0.227
HbA1c (mmol/mol)	36.2 (4.6)	36.3 (4.1)	36.9 (4.2)	0.147	60.9 (11.1)	57.5 (8.7)	59.7 (9.8)	0.149	60.7 (16.7)	63.6 (16.1)	61.0 (14.4)	0.468
HbA1c (%)	5.46 (0.42)	5.47 (0.38)	5.53 (0.38)	0.174	7.73 (1.01)	7.41 (0.80)	7.61 (0.89)	0.149	7.72 (1.52)	7.97 (1.47)	7.74 (1.32)	0.494
Total cholesterol (mg/dL)	196.0 (36.0)	203.0 (35.6)	203.0 (38.2)	0.070	176.0 (31.3)	182.0 (27.9)	185.0 (25.6)	0.122	192.0 (36.5)	180.0 (35.7)	185.0 (38.1)	0.151
HDL-cholesterol (mg/dL)	58.9 (15.9)	59.6 (14.1)	61.3 (14.9)	0.217	62.0 (17.2)	65.1 (16.4)	66.5 (13.7)	0.174	49.3 (14.9)	48.6 (11.5)	51.3 (13.1)	0.364
LDL-cholesterol (mg/dL)	117.0 (30.3)	123.0 (31.1)	123.0 (32.8)	0.042	99.8 (25.2)	102.0 (23.4)	105.0 (22.4)	0.363	114.0 (28.7)	103.0 (30.7)	110.0 (32.6)	0.087
Triglycerides (mg/dL)	108.0 (90.0)	103.0 (55.4)	97.2 (53.4)	0.303	73.5 (32.9)	71.9 (24.7)	78.8 (53.0)	0.554	148.0 (149)	153.0 (100)	125.0 (64.4)	0.173

Data are shown as n (%) for categorical variables and mean (SD) for continuous variables. BMI, body mass index; DBP, diastolic blood pressure; HbA1c, glycated hemoglobin; HDL-cholesterol, high density lipoprotein-cholesterol; LDL-cholesterol, low density lipoprotein-cholesterol; SBP, systolic blood pressure.

P was calculated according to the method of Benjamini and Hochberg for multiple comparisons.

Table S2: Clinical characteristics and the alternate Healthy Eating Index (aHEI) by population

Characteristics	Controls				T1DM				T2DM			
	T1 [20-38] (N= 371)	T2 [38-44] (N= 110)	T3 [44-68] (N=187)	p	T1 [20-38] (N= 90)	T2 [38-44] (N= 54)	T3 [44-68] (N=78)	p	T1 [20-38] (N= 79)	T2 [38-44] (N= 70)	T3 [44-68] (N=103)	p
Age (years)	47.0 (12.3)	49.0 (12.6)	54.5 (13.4)	<0.001	41.8 (9.8)	43.0 (9.7)	48.8 (11.2)	<0.001	55.8 (10.3)	58.0 (9.4)	59.8 (9.1)	0.050
Sex (women)	157 (50.6)	128 (60.4)	106 (72.6)	<0.001	36 (49.3)	38 (49.4)	45 (62.5)	0.183	13 (30.2)	37 (46.2)	71 (55.0)	0.017
Educational level				0.678				0.350				0.812
Undergraduate	252 (81.6)	172 (81.9)	123 (84.8)		52 (77.6)	49 (67.1)	53 (74.6)		38 (90.5)	72 (93.5)	114 (91.9)	
Graduate or higher	57 (18.4)	38 (18.1)	22 (15.2)		15 (22.4)	24 (32.9)	18 (25.4)		4 (9.5)	5 (6.3)	10 (7.8)	
Tobacco exposure	195 (62.9)	99 (46.7)	54 (36.9)	<0.001	36 (49.3)	40 (52.0)	36 (50.0)	0.450	32 (74.4)	45 (56.3)	59 (45.7)	0.001
Regular physical activity	184 (61.1)	155 (73.8)	109 (75.7)	0.001	50 (69.4)	52 (67.5)	59 (81.9)	0.104	20 (46.5)	42 (52.5)	58 (45.0)	0.563
BMI (Kg/m2)	26.0 (4.51)	26.3 (4.6)	26.1 (4.2)	0.646	25.9 (4.4)	25.6 (4.3)	25.4 (3.3)	0.797	31.1 (4.9)	32.7 (6.1)	31.2 (5.2)	0.136
Waist (cm)	93.3 (13.0)	93.8 (12.1)	92.7 (11.4)	0.693	89.3 (12.3)	89.4 (12.4)	87.2 (11.7)	0.486	105 (10.6)	108 (12.3)	104 (11.6)	0.038
SBP (mmHg)	122.0 (15.4)	123.0 (17.6)	123.0 (16.7)	0.666	126.0 (19.2)	126.0 (15.5)	127.0 (18.2)	0.830	137.0 (20.1)	140.0 (20.0)	139.0 (17.4)	0.635
DBP (mmHg)	76.5 (9.0)	77.4 (10.4)	76.7 (10.4)	0.561	74.1 (9.5)	75.0 (9.1)	73.2 (10.4)	0.502	77.5 (9.4)	78.5 (10.3)	74.9 (10.7)	0.041
Hypertension	36 (11.6)	29 (13.7)	34 (23.3)	0.004	13 (17.8)	20 (26.0)	22 (30.6)	0.197	22 (51.2)	46 (57.5)	73 (56.6)	0.779
Dyslipidemia	52 (16.8)	37 (17.5)	48 (32.9)	<0.001	26 (35.6)	33 (42.9)	32 (44.4)	0.512	18 (41.9)	36 (45.0)	66 (51.2)	0.486
HbA1c (mmol/mol)	36.0 (4.40)	36.7 (4.4)	36.8 (4.4)	0.121	61.4 (11.2)	58.0 (8.8)	59.7 (10.2)	0.110	57.5 (15.0)	60.4 (15.8)	63.8 (15.4)	0.053
HbA1c (%)	5.5 (0.4)	5.5 (0.4)	5.5 (0.4)	0.130	7.8 (1.0)	7.5 (0.1)	7.6 (0.9)	0.110	7.4 (1.4)	7.7 (1.5)	8.0 (1.4)	0.065
Total cholesterol (mg/dL)	197.0 (36.0)	197.0 (36.0)	207.0 (38.3)	0.023	177.0 (27.6)	182.0 (30.2)	182.0 (28.2)	0.399	187.0 (36.3)	181.0 (39.1)	188.0 (36.1)	0.481
HDL-cholesterol (mg/dL)	59.4 (15.8)	59.7 (15.5)	60.3 (14.1)	0.858	61.3 (15.6)	64.9 (17.5)	66.9 (14.2)	0.104	50.4 (16.6)	49.1 (13.6)	50.3 (11.9)	0.813
LDL-cholesterol (mg/dL)	118.0 (30.8)	119.0 (31.0)	126.0 (32.2)	0.048	101.0 (23.8)	103.0 (24.9)	102.0 (22.9)	0.842	110.0 (26.6)	105.0 (33.2)	112.0 (31.1)	0.344
Triglycerides (mg/dL)	108.0 (94.9)	97.0 (56.5)	105.0 (53.0)	0.241	73.8 (27.4)	76.9 (47.5)	74.1 (41.3)	0.868	149.0 (193)	141.0 (95.0)	136.0 (69.4)	0.773

Data are shown as n (%) for categorical variables and mean (SD) for continuous variables. BMI, body mass index; DBP, diastolic blood pressure; HbA1c, glycated hemoglobin; HDL-cholesterol, high density lipoprotein-cholesterol; LDL-cholesterol, low density lipoprotein-cholesterol; SBP, systolic blood pressure.

P was calculated according to the method of Benjamini and Hochberg for multiple comparisons.

Table S3: Daily nutrient intake by the alternate Mediterranean Diet score and the alternate Healthy Eating Index

Nutrients (units/day)	aMED				aHEI			
	T1 [0-4) (N= 540)	T2 [4] (N= 234)	T3 [5-9] (N=368)	p	T1 [20-38) (N = 426)	T2 [38-44) (N=369)	T3 [44-68] (N=347)	p
**Energy intake (kcal)**	**2116 (585)**	2179 (610)	2161 (534)	0.247	2166 (602)	2136 (591)	2125 (521)	0.316
Proteins (g)	96.5 (14.4)	97.0 (17.3)	98.5 (18.7)	0.101	94.1 (13.9)	96.3 (14.6)	102 (20.0)	<0.001
Carbohydrates (g)	206 (38.0)	206 (43.6)	207 (35.1)	0.883	199 (38.1)	204 (36.3)	218 (37.9)	<0.001
Complex carbohydrates (g)	90.9 (21.2)	87.4 (21.1)	88.2 (19.5)	0.059	89.3 (21.7)	87.6 (19.6)	91.1 (20.5)	0.276
Sugar (g)	80.5 (27.9)	84.7 (35.0)	84.9 (24.7)	0.030	76.9 (26.6)	81.7 (25.7)	91.3 (31.8)	<0.001
Added sugar (g)	26.2 (19.7)	22.6 (26.2)	18.7 (15.9)	<0.001	26.9 (20.0)	21.9 (17.8)	19.5 (22.7)	<0.001
Gycemic load (%)	96.1 (21.6)	91.9 (23.0)	89.1 (18.5)	<0.001	92.7 (22.8)	91.8 (20.1)	94.7 (20.1)	0.269
Total fat (g)	93.4 (17.2)	93.9 (17.7)	92.9 (15.2)	0.765	94.5 (17.2)	96.0 (16.6)	89.2 (15.2)	<0.001
Saturated fat (g)	26.8 (5.29)	24.7 (5.03)	23.6 (4.81)	<0.001	27.1 (5.41)	25.7 (4.63)	22.8 (4.82)	<0.001
Monounsaturated fat (g)	44.6 (11.9)	46.9 (12.9)	46.6 (10.9)	0.017	45.7 (12.2)	47.9 (11.8)	43.5 (11.0)	0.027
Polyunsaturated fat (g)	15.1 (4.69)	15.6 (4.04)	16.2 (4.35)	0.002	14.9 (3.85)	15.7 (4.44)	16.2 (5.08)	<0.001
Omega 3 (g)	1.47 (0.32)	1.65 (0.57)	1.74 (0.52)	<0.001	1.44 (0.27)	1.60 (0.35)	1.78 (0.65)	<0.001
Omega 6 (g)	13.5 (4.66)	13.8 (3.97)	14.3 (4.27)	0.017	13.3 (3.79)	14.0 (4.41)	14.3 (5.03)	0.003
Daily food intake (g/day)								
Dairy products	360.0 (215.0)	351.0 (245.0)	330.0 (218.0)	0.051	347.0 (223.0)	347.0 (216.0)	352.0 (229.0)	0.774
Eggs	21.7 (18.3)	21.6 (16.7)	19.5 (11.9)	0.049	21.1 (12.4)	21.7 (21.3)	20.2 (13.9)	0.436
White meat	34.6 (17.9)	39.1 (23.1)	40.4 (35.2)	0.001	30.9 (16.9)	36.5 (16.6)	46.3 (37.7)	<0.001
Red meat	63.7 (33.1)	49.5 (28.6)	44.2 (25.4)	<0.001	63.5 (34.2)	56.0 (28.0)	41.7 (25.8)	<0.001
Processed meat	41.5 (27.8)	35.2 (21.9)	28.3 (20.0)	<0.001	43.3 (27.2)	35.2 (23.5)	27.7 (20.6)	<0.001
Meat	140.0 (46.5)	124.0 (45.1)	112.0 (46.4)	<0.001	138.0 (49.3)	128.0 (39.7)	115.0 (50.6)	<0.001
Lean fish	24.7 (19.1)	35.1 (22.4)	40.4 (34.9)	<0.001	23.6 (18.5)	31.7 (19.7)	42.2 (36.5)	<0.001
Fatty fish	24.8 (21.3)	35.2 (50.2)	42.2 (53.2)	<0.001	23.1 (17.3)	30.5 (25.6)	46.3 (65.0)	<0.001
Seafood	10.4 (9.8)	12.0 (10.7)	12.6 (9.7)	0.001	10.6 (7.79)	11.5 (12.2)	12.3 (9.72)	0.015
Fish	59.8 (34.5)	82.2 (59.5)	95.1 (70.4)	<0.001	57.2 (30.3)	73.7 (36.2)	101.0 (82.0)	<0.001
Fruits and vegetables	381.0 (192.0)	545.0 (227.0)	611.0 (223.0)	<0.001	318.0 (127.0)	498.0 (166.0)	689.0 (236.0)	<0.001
Nuts	5.3 (7.3)	8.3 (8.8)	14.7 (15.8)	<0.001	5.4 (6.7)	7.9 (9.0)	14.4 (16.4)	<0.001

Legumes	21.3 (17.9)	24.3 (20.7)	38.1 (35.8)	<0.001	24.1 (19.6)	26.5 (22.6)	32.1 (35.9)	<0.001
Cereal and pasta	70.2 (42.8)	72.8 (45.9)	72.7 (47.5)	0.390	70.8 (41.1)	71.2 (44.8)	72.8 (49.5)	0.546
Potatoes	52.8 (34.6)	54.9 (58.9)	54.1 (37.7)	0.618	51.4 (49.8)	55.2 (34.8)	54.8 (37.1)	0.242
Bred	119.0 (71.9)	102.0 (54.9)	98.5 (58.9)	<0.001	116.0 (71.4)	106.0 (62.6)	104.0 (59.6)	0.008
Sweets	38.0 (37.8)	35.0 (39.7)	31.1 (31.4)	0.005	37.9 (40.1)	35.7 (35.1)	31.2 (32.4)	0.012
Vegetable fats	28.5 (19.1)	32.3 (18.8)	33.2 (18.1)	<0.001	29.9 (19.1)	34.5 (20.2)	28.0 (16.3)	0.268
Animal fats	0.4 (1.6)	0.3 (1.0)	0.4 (2.0)	0.891	0.4 (1.9)	0.3 (0.8)	0.4 (2.0)	0.469
Alcohol drinks	102.0 (180.0)	117.0 (163.0)	112.0 (141.0)	0.325	162.0 (223.0)	86.7 (117.0)	66.3 (93.4)	<0.001
Non-alcoholic beverages	1366.0 (547.0)	1356.0 (495.0)	1406.0 (574.0)	0.309	1355.0 (559)	1403 (506)	1376 (571)	0.548
Coffee and tea	393 (297)	408 (313)	378 (275)	0.527	395.0 (291.0)	410.0 (303.0)	366.0 (286.0)	0.204
Prepared meals	80.8 (75.3)	86.9 (119)	77.2 (64.7)	0.599	72.2 (63.6)	81.7 (94.2)	90.6 (90.6)	0.002
Salt	0.60 (0.77)	0.58 (0.74)	0.45 (0.65)	0.003	0.70 (0.82)	0.59 (0.74)	0.32 (0.52)	<0.001

Data are shown as n (%) for categorical variables and mean (SD) for continuous variables.

P was calculated according to the method of Benjamini and Hochberg for multiple comparisons.

Table S4: The advanced lipoprotein profile and the alternate Mediterranean Diet score (aMED) by population

NMR variable	Controls				T1DM				T2DM			
	T1 [0,4) (N= 371)	T2 [4] (N= 110)	T3 [5,9] (N=187)	p	T1 [0,4) (N= 90)	T2 [4] (N= 54)	T3 [5,9] (N=78)	p	T1 [0,4) (N= 79)	T2 [4] (N= 70)	T3 [5,9] (N=103)	p
VLDL-P number (nmol/L)												
Total	49.3 (38.0)	45.5 (28.8)	41.4 (24.7)	0.021	32.2 (15.0)	29.6 (12.0)	33.6 (27.3)	0.750	71.3 (62.2)	80.0 (55.3)	60.9 (36.7)	0.609
Large	1.2 (0.8)	1.1 (0.6)	1.0 (0.5)	0.012	0.9 (0.4)	0.8 (0.3)	0.9 (0.5)	0.750	1.7 (1.36)	1.9 (1.09)	1. 5 (0.7)	0.609
Medium	5.0 (5.7)	4. 6 (3.7)	4.2 (2.4)	0.055	3.1 (1.7)	3.2 (1. 6)	3.7 (3.9)	0.486	6.8 (9.6)	7.3 (8.7)	5.5 (3.9)	0.624
Small	43.1 (32.6)	39.9 (25.1)	36.3 (22.3)	0.021	28.2 (13.1)	25.6 (10.5)	29.1 (23.1)	0.771	62.9 (51.9)	70.9 (47.0)	53.9 (32.6)	0.609
VLDL-P composition (mg/dL)												
VLDL-C	12.0 (11.1)	11.0 (9.0)	10.0 (7.7)	0.039	7.9 (5.9)	7.3 (5.4)	8.4 (9.5)	0.750	17.8 (16.6)	19.6 (14.7)	15.2 (11.7)	0.624
VLDL-TG	71.4 (57.6)	65.6 (41.6)	59.4 (33.9)	0.021	45.9 (20.5)	42.4 (15.6)	48.4 (39.5)	0.750	102 (98.3)	115 (85.6)	86.6 (50.8)	0.609
VLDL-P size (nm)	42.1 (0.2)	42.1 (0.2)	42.1 (0.2)	0.430	42.1 (0.2)	42.2 (0.3)	42.1 (0.23)	0.619	42.0 (0.20)	42.0 (0.24)	42.0 (0.20)	0.624
LDL-P number (nmol/L)												
Total	1307.0 (270.0)	1383.0 (276.0)	1377.0 (296.0)	0.012	1249.0 (196.0)	1274.0 (210.0)	1284.0 (190.0)	0.486	1339.0 (249.0)	1269.0 (263.0)	1327.0 (275.0)	0.905
Large	184.0 (34.2)	192.0 (37.7)	195.0 (38.8)	0.006	180.0 (30.1)	184.0 (29.3)	183.0 (30.0)	0.583	179.0 (33.8)	165.0 (34.7)	175.0 (33.1)	0.835
Medium	411.0 (134.0)	453.0 (143.0)	462.0 (154.0)	0.001	404.0 (110.0)	424.0 (106.0)	422.0 (110.0)	0.502	402.0 (137.0)	352.0 (130.0)	394.0 (134.0)	0.887
Small	711.0 (147.0)	738.0 (140.0)	720.0 (143.0)	0.460	664.0 (86.4)	666.0 (103.0)	678.0 (93.5)	0.516	758.0 (129.0)	751.0 (131.0)	759.0 (141.0)	0.941
LDL-P composition (mg/dL)												
LDL-C	127.0 (26.7)	136.0 (27.6)	135.0 (29.8)	0.006	123.0 (20.2)	126.0 (20.6)	126.0 (20.5)	0.513	127.0 (25.1)	118.0 (25.7)	125.0 (27.4)	0.887
LDL-TG	16.3 (5.0)	17.2 (5.4)	17.7 (5.4)	0.008	15.6 (4.6)	16.4 (4.5)	16.6 (4.0)	0.486	18.0 (5.4)	17.0 (5.4)	17.7 (4.8)	0.887
LDL-P size (nm)	21.0 (0.27)	21.0 (0.26)	21.1 (0.26)	0.012	21.0 (0.2)	21.1 (0.2)	21.1 (0.2)	0.750	20.9 (0.4)	20.8 (0.3)	20.8 (0.2)	0.835
HDL-P number (µmol/L)												
Total	28.9 (5.7)	29.8 (5.7)	29.7 (5.3)	0.181	31.2 (6.5)	32.9 (6.7)	33.0 (4.9)	0.397	27.1 (6.2)	26.5 (4.7)	28.0 (5.5)	0.624

Large	0.3 (0.1)	0.3 (0.1)	0.3 (0.1)	0.055	0.3 (0.1)	0.3 (0.1)	0.3 (0.1)	0.397	0.26 (0.06)	0.26 (0.04)	0.27 (0.04)	0.814
Medium	9.4 (2.2)	9.4 (2.1)	9.7 (2.0)	0.276	10.5 (2.6)	11.0 (2.9)	11.1 (2.3)	0.486	8.1 (1.9)	8.0 (1.5)	8.5 (1.9)	0.609
Small	19.3 (4.2)	20.1 (4.4)	19.7 (3.8)	0.216	20.4 (4.5)	21.6 (4.4)	21.6 (3.5)	0.397	18.7 (5.1)	18.2 (3.8)	19.2 (4.4)	0.828
HDL-P composition (mg/dL)												
HDL-C	56.0 (12.7)	57.3 (12.1)	57.9 (11.4)	0.119	62.5 (15.3)	66.3 (15.1)	66.0 (11.8)	0.486	49.5 (12.2)	48.0 (9.5)	51.9 (11.4)	0.609
HDL-TG	13.6 (4.7)	13.8 (4.5)	13.6 (4.7)	0.909	13.5 (3.6)	13.9 (4.0)	14.8 (4.2)	0.397	14.0 (5.6)	14.3 (4.0)	14.1 (4.5)	0.887
HDL-P size (nm)	8.2 (0.1)	8.2 (0.1)	8.2 (0.1)	0.909	8.2 (0.1)	8.2 (0.1)	8.2 (0.1)	0.892	8.2 (0.1)	8.2 (0.1)	8.2 (0.1)	0.887
IDL-P composition (mg/dL)												
IDL-C	9.9 (4.6)	10.6 (4.7)	10.5 (4.4)	0.169	123.0 (20.2)	126.0 (20.6)	126.0 (20.5)	0.513	13.2 (5.3)	13.7 (5.4)	13.0 (4.9)	0.887
IDL-TG	16.3 (5.0)	17.2 (5.4)	17.7 (5.4)	0.008	10.7 (3.2)	11.1 (3.3)	11.5 (4.0)	0.486	14.6 (4.3)	15.1 (4.6)	14.5 (4.1)	0.887
Other atherogenic variables												
Non-HDL-P (nmol/L)	1327.0 (281.0)	1399.0 (282.0)	1388.0 (301.0)	0.022	1249.0 (201.0)	1271.0 (217.0)	1284.0 (191.0)	0.486	1383.0 (238.0)	1322.0 (270.0)	1360.0 (280.0)	0.887
Total-P/HDL-P	48.6 (13.7)	49.9 (14.1)	49.4 (14.1)	0.540	42.8 (11.8)	41.6 (13.7)	40.8 (8.5)	0.486	54.7 (15.0)	52.6 (14.9)	51.8 (15.9)	0.624
LDL-P/HDL-P	46.8 (13.0)	48.3 (13.5)	47.9 (13.7)	0.416	41.7 (11.4)	40.7 (13.3)	39.7 (8.2)	0.486	51.9 (14.5)	49.3 (13.6)	49.5 (15.1)	0.624

Data are shown as mean (SD). HDL high-density lipoprotein, HDL-C cholesterol content in HDL, HDL-P HDL particles, HDL-TG triglyceride content in HDL, IDL intermediate-density lipopro-tein, IDL-C cholesterol content in IDL, IDL-TG triglyceride content in IDL, LDL low-density lipo-protein, LDL-C cholesterol content in LDL, LDL-P LDL particles, LDL-TG triglyceride content in LDL, NMR nuclear magnetic resonance, VLDL very lowdensity lipoprotein, VLDL-C cholesterol content in VLDL, VLDL-P VLDL particles, VLDL-TG triglyceride content in VLD. T1=poor adherence, T2=moderate adherence, and T3=high adherance

Table S5: The advanced lipoprotein profile and the alternate Healthy Eating Index (aHEI) scores stratified by study group

NMR variable	Controls T1 [20,38) (N = 310)	Controls T2 [38,44) (N=212)	Controls T3 [44,68] (N=146)	p	T1DM T1 [20,38) (N = 73)	T1DM T2 [38,44) (N=77)	T1DM T3 [44,68] (N=72)	p	T2DM T1 [20,38) (N = 43)	T2DM T2 [38,44) (N=80)	T2DM T3 [44,68] (N=129)	p
VLDL-P number (nmol/L)												
Total	48.6 (39.0)	43.9 (28.8)	45.7 (26.3)	0.335	32.5 (12.9)	32.9 (24.6)	30.7 (19.7)	0.946	70.6 (75.5)	70.7 (51.3)	68.3 (41.3)	0.868
Large	1.2 (0.8)	1.0 (0.6)	1.0 (0.6)	0.280	0.9 (0.3)	0.9 (0.5)	0.8 (0.4)	0.729	1.7 (1.7)	1.7 (1.0)	1.6 (0.8)	0.829
Medium	5.1 (6.3)	4.4 (2.7)	4.5 (2.4)	0.335	3. 5 (1.8)	3.3 (3.1)	3.2 (2.9)	0.946	6.8 (12.4)	6.7 (8.3)	6.0 (4.0)	0.829
Small	42.4 (32.9)	38.4 (26.0)	40.1 (23.7)	0.357	28.2 (11.0)	28.8 (21.2)	26.6 (16.6)	0.946	62.1 (62.0)	62.3 (43.1)	60.7 (37.0)	0.879
VLDL-P composition (mg/dL)												
VLDL-C	11.8 (11.1)	10.7 (9.0)	11.2 (8.5)	0.474	8.4 (6.0)	7.9 (8.4)	7.4 (7.2)	0.928	17.3 (19.6)	17.3 (14.0)	17.2 (12.5)	0.987
VLDL-TG	70.5 (60.5)	63.1 (39.4)	65.5 (35.5)	0.335	46.5 (17.2)	47.1 (34.8)	44.1 (28.5)	0.946	102.0 (122.0)	102.0 (80.2)	96.7 (57.3)	0.843
VLDL-P size (nm)	42.1 (0.2)	42.1 (0.2)	42.1 (0.2)	0.335	42.2 (0.2)	42.1 (0.2)	42.1 (0.2)	0.826	42.0 (0.2)	42.0 (0.2)	42.0 (0.2)	0.899
LDL-P number (nmol/L)												
Total	1321.0 (276.0)	1332.0 (277.0)	1386.0 (293.0)	0.100	1262.0 (209.0)	1273.0 (197.0)	1266.0 (187.0)	0.994	1285.0 (250.0)	1294.0 (274.0)	1337.0 (263.0)	0.699
Large	186.0 (35.4)	187.0 (34.9)	197.0 (39.3)	0.036	184.0 (31.2)	180.0 (28.2)	182.0 (30.3)	0.994	175.0 (35.5)	169.0 (35.8)	176.0 (32.4)	0.829
Medium	420.0 (140.0)	428.0 (140.0)	464.0 (148.0)	0.036	416.0 (116.0)	415.0 (101.0)	415.0 (111.0)	0.994	379.0 (122.0)	369.0 (139.0)	396.0 (137.0)	0.699
Small	716.0 (148.0)	717.0 (139.0)	725.0 (145.0)	0.561	662.0 (89.9)	677.0 (102.0)	669.0 (85.8)	0.946	731.0 (142.0)	756.0 (137.0)	765.0 (130.0)	0.699
LDL-P composition (mg/dL)												
LDL-C	129.0 (27.2)	130.0 (27.9)	136.0 (29.2)	0.065	125.0 (21.2)	125.0 (19.8)	125.0 (20.4)	0.994	122.0 (24.5)	121.0 (27.6)	126.0 (26.2)	0.699
LDL-TG	16.5 (5.3)	16.5 (4.8)	18.0 (5.4)	0.060	16.1 (5.0)	16.1 (4.2)	16.2 (3.9)	0.994	17.0 (4.9)	17.1 (5.5)	18.1 (5.0)	0.699
LDL-P size (nm)	21.0 (0.3)	21.0 (0.3)	21.1 (0.3)	0.065	21.1 (0.2)	21.0 (0.2)	21.1 (0.3)	0.946	20.9 (0.4)	20.8 (0.3)	20.8 (0.2)	0.841

HDL-P number (µmol/L)												
Total	29.1 (5.9)	29.1 (5.3)	29.9 (5.6)	0.335	31.0 (5.4)	32.1 (7.3)	33.8 (4.8)	0.046	26.9 (7.0)	26.8 (5.3)	27.7 (5.2)	0.699
Large	0.3 (0.1)	0.3 (0.1)	0.3 (0.1)	0.018	0.3 (0.1)	0.3 (0.1)	0.30 (0.04)	0.033	0.3 (0.1)	0.3 (0.0)	0.3 (0.0)	0.829
Medium	9.3 (2.2)	9.5 (2.1)	9.8 (2.1)	0.091	10.3 (2.4)	10.8 (3.1)	11.5 (2.0)	0.046	8.2 (2.3)	8.2 (1.7)	8.3 (1.7)	0.868
Small	19.6 (4.4)	19.3 (3.8)	19.8 (4.1)	0.714	20.4 (3.9)	21.1 (4.8)	21.9 (3.6)	0.099	18.4 (5.5)	18.4 (4.4)	19.1 (4.2)	0.699
HDL-P composition (mg/dL)												
HDL-C	56.2 (12.7)	56.7 (12.0)	58.0 (11.8)	0.335	61.7 (12.8)	64.5 (17.1)	67.9 (11.2)	0.056	50.0 (13.3)	49.1 (11.0)	50.6 (10.7)	0.840
HDL-TG	13.6 (4.6)	13.3 (4.8)	14.3 (4.6)	0.335	13.4 (3.4)	13.8 (4.3)	15.0 (4.0)	0.096	13.4 (7.2)	14.1 (3.7)	14.4 (4.3)	0.699
HDL-P size (nm)	8.2 (0.1)	8.2 (0.1)	8.2 (0.1)	0.909	8.2 (0.1)	8.2 (0.1)	8.2 (0.1)	0.729	8.2 (0.1)	8.2 (0.1)	8.2 (0.1)	0.716
IDL-P composition (mg/dL)												
IDL-C	9.9 (4.6)	10.6 (4.7)	10.5 (4.4)	0.169	9.9 (4.9)	9.6 (4.6)	10.0 (4.1)	0.994	12.2 (4.6)	13.2 (5.2)	13.7 (5.3)	0.699
IDL-TG	16.3 (5.0)	17.2 (5.4)	17.7 (5.4)	0.008	11.1 (3.5)	11.0 (3.7)	11.2 (3.5)	0.994	13.6 (4.0)	14.4 (4.3)	15.2 (4.3)	0.652
Other atherogenic variables												
Non-HDL-P (nmol/L)	1327.0 (281.0)	1399.0 (282.0)	1388.0 (301.0)	0.022	1264.0 (213.0)	1273.0 (206.0)	1263.0 (187.0)	0.994	1329.0 (242.0)	1337.0 (276.0)	1378.0 (265.0)	0.699
Total-P/HDL-P	48.6 (13.7)	49.9 (14.1)	49.4 (14.1)	0.540	43.1 (11.1)	43.0 (13.8)	39.1 (7.5)	0.109	53.8 (16.8)	52.8 (14.8)	52.7 (15.2)	0.868
LDL-P/HDL-P	46.8 (13.0)	48.3 (13.5)	47.9 (13.7)	0.416	42.0 (10.7)	41.9 (13.3)	38.2 (7.4)	0.109	51.1 (16.5)	49.9 (13.7)	50.1 (14.4)	0.868

Data are shown as mean (SD). HDL high-density lipoprotein, HDL-C cholesterol content in HDL, HDL-P HDL particles, HDL-TG triglyceride content in HDL, IDL intermediate-density lipopro-tein, IDL-C cholesterol content in IDL, IDL-TG triglyceride content in IDL, LDL low-density lipo-protein, LDL-C cholesterol content in LDL, LDL-P LDL particles, LDL-TG triglyceride content in LDL, NMR nuclear magnetic resonance, VLDL very lowdensity lipoprotein, VLDL-C cholesterol content in VLDL, VLDL-P VLDL particles, VLDL-TG triglyceride content in VLD. T1=poor adherence, T2=moderate adherence, and T3=high adherance

Table S6: The advanced lipoprotein profile, the alternate Mediterranean Diet score and the alternate Healthy Eating Index by age groups

	aMED								aHEI							
	<50 years				>50 years				<50 years				>50 years			
**Characteristics**	**T1 [0-4] (N= 173)**	**T2 [4] (N= 55)**	**T3 [5-9] (N=90)**	**P**	**T1 [0-4] (N = 140)**	**T2 [4] (N=109)**	**T3 [5-9] (N=69)**	**p**	**T1 [20-38] (N= 118)**	**T2 [38-44] (N= 67)**	**T3 [44-68] (N=128)**	**P**	**T1 [20-38] (N = 66)**	**T2 [38-44] (N=94)**	**T3 [44-68] (N=153)**	**p**
VLDL-P number (nmol/L)																
Total	39.4 (27.2)	36.4 (20.2)	35.0 (23.5)	0.210	36.6 (23.4)	38.7 (27.2)	38.1 (25.2)	0.775	47.7 (25.2)	60.8 (42.9)	44.4 (27.5)	0.868	45.1 (26.1)	46.0 (28.2)	52.9 (34.5)	0.322
Large	1.0 (0.6)	0.9 (0.4)	0.9 (0.5)	0.086	0.9 (0.5)	1.0 (0.6)	1.0 (0.5)	0.775	1.1 (0.5)	1.4 (0.9)	1.1 (0.6)	0.868	1.1 (0.5)	1.1 (0.6)	1.25 (0.7)	0.322
Medium	3.98 (2.8)	3.56 (2.0)	3.3 (2.2)	0.072	3.8 (2.4)	3.7 (2.7)	3.6 (2.4)	0.775	4.42 (2.56)	5.53 (4.36)	4.37 (3.03)	0.957	4.2 (2.4)	4.6 (3.4)	4.9 (3.4)	0.378
Small	34.5 (24.3)	31.9 (18.2)	30.9 (21.1)	0.236	31.9 (20.9)	34.1 (24.3)	33.5 (22.6)	0.775	42.2 (22.7)	53.9 (38.2)	38.9 (24.3)	0.862	39.8 (23.7)	40.3 (24.7)	46.8 (30.9)	0.322
VLDL-P composition (mg/dL)																
VLDL-C	9.56 (8.8)	8.59 (7.2)	8.6 (7.2)	0.357	8.9 (8.0)	9.2 (8.4)	9.3 (8.0)	0.775	11.9 (8.29)	15.1 (12.2)	10.6 (8.68)	0.862	11.0 (8.4)	11.0 (8.4)	13.1 (10.6)	0.325
VLDL-TG	56.8 (37.5)	52.1 (26.9)	49.4 (32.3)	0.164	52.7 (32.0)	55.3 (37.7)	54.1 (34.5)	0.775	67.6 (34.1)	86.5 (60.8)	64.0 (38.5)	0.868	64.0 (35.1)	66.3 (40.9)	75.2 (47.9)	0.322
VLDL-P size (nm)	42.1 (0.2)	42.1 (0.2)	42.1 (0.2)	0.027	42.2 (0.2)	42.1 (0.2)	42.1 (0.2)	0.085	42.1 (0.22)	42.0 (0.22)	42.1 (0.23)	0.862	42.1 (0.2)	42.1 (0.2)	42.1 (0.2)	0.473
LDL-P number (nmol/L)																
Total	1204.0 (252.0)	1290.0 (241.0)	1344.0 (304.0)	<0.001	1218.0 (255.0)	1284.0 (284.0)	1302.0 (277.0)	0.128	1398 (260)	1386 (243)	1366 (245)	0.862	1428.0 (249.0)	1351.0 (253.0)	1382.0 (247.0)	0.583
Large	178.0 (31.9)	189.0 (35.7)	195.0 (37.9)	0.001	182.0 (33.6)	185.0 (36.7)	189.0 (35.4)	0.323	195 (36.2)	187 (37.1)	190 (34.6)	0.862	201.0 (38.1)	186.0 (34.7)	190.0 (34.8)	0.325
Medium	386.0 (124.0)	438.0 (123.0)	470.0 (155.0)	<0.001	405.0 (131.0)	426.0 (150.0)	437.0 (133.0)	0.303	466 (135)	445 (132)	457 (132)	0.873	492.0 (141.0)	436.0 (125.0)	456.0 (132.0)	0.384
Small	640.0 (131.0)	663.0 (112.0)	679.0 (141.0)	0.050	631.0 (122.0)	673.0 (132.0)	676.0 (141.0)	0.107	737 (131)	754 (123)	720 (117)	0.862	735.0 (109.0)	728.0 (134.0)	736.0 (125.0)	0.907
LDL-P composition (mg/dL)																
LDL-C	119.0 (24.8)	128.0 (24.1)	133.0 (30.6)	<0.001	121.0 (25.5)	126.0 (29.0)	129.0 (26.9)	0.155	136 (26.1)	133 (25.0)	133 (25.7)	0.862	141.0 (25.8)	131.0 (25.1)	134.0 (25.7)	0.378
LDL-TG	15.3 (5.0)	17.1 (5.1)	17.9 (5.4)	<0.001	15.9 (5.3)	16.6 (5.3)	16.9 (5.1)	0.323	18.5 (5.1)	18.8 (5.1)	18.4 (4.3)	0.957	19.0 (5.4)	17.6 (4.8)	18.9 (4.4)	0.834
LDL-P size (nm)	21.1 (0.3)	21.1 (0.2)	21.2 (0.2)	0.028	21.1 (0.2)	21.1 (0.2)	21.1 (0.2)	0.562	21.0 (0.3)	21.0 (0.3)	21.0 (0.3)	0.957	21.1 (0.3)	21.0 (0.25)	21.0 (0.26)	0.325

HDL-P number (µmol/L)																
Total	30.4 (6.0)	32.6 (6.7)	31.2 (5.5)	0.231	30.6 (6.3)	30.9 (6.0)	32.0 (5.3)	0.323	31.1 (6.2)	31.5 (5.3)	31.4 (5.0)	0.905	31.5 (5.5)	31.6 (6.0)	31.1 (5.3)	0.724
Large	0.3 (0.1)	0.3 (0.1)	0.3 (0.1)	0.007	0.3 (0.1)	0.3 (0.1)	0.3 (0.1)	0.128	0.30 (0.1)	0.3 (0.0)	0.3 (0.0)	0.868	0.3 (0.1)	0.3 (0.1)	0.3 (0.0)	0.907
Medium	10.3 (2.3)	11.0 (2.9)	10.7 (2.2)	0.177	10.3 (2.4)	10.5 (2.4)	10.8 (2.2)	0.323	10.1 (2.5)	9.71 (2.0)	10.1 (2.1)	0.957	10.1 (2.1)	10.2 (2.6)	9.87 (2.1)	0.583
Small	19.9 (4.3)	21.3 (4.3)	20.2 (3.8)	0.357	20.0 (4.4)	20.1 (4.1)	20.9 (3.8)	0.323	20.7 (4.3)	21.5 (3.9)	21.1 (3.8)	0.868	21.2 (4.1)	21.1 (4.2)	20.9 (4.0)	0.834
HDL-P composition (mg/dL)																
HDL-C	59.7 (13.4)	64.4 (15.8)	61.9 (11.6)	0.177	60.3 (13.8)	61.0 (14.2)	63.0 (11.2)	0.323	60.5 (14.7)	59.1 (12.8)	60.7 (11.8)	0.957	61.0 (12.8)	61.7 (14.8)	59.2 (12.2)	0.459
HDL-TG	14.4 (4.6)	15.1 (4.2)	14.6 (4.9)	0.645	14.3 (4.7)	14.5 (4.3)	15.2 (5.0)	0.337	14.7 (4.0)	16.0 (3.4)	14.9 (3.8)	0.873	14.9 (4.2)	14.5 (3.1)	15.4 (4.0)	0.444
HDL-P size (nm)	8.2 (0.1)	8.2 (0.1)	8.3 (0.1)	0.563	8.2 (0.1)	8.2 (0.1)	8.2 (0.1)	0.855	8.2 (0.1)	8.2 (0.1)	8.2 (0.1)	0.868	8.2 (0.1)	8.2 (0.1)	8.2 (0.1)	0.834
IDL-P composition (mg/dL)																
IDL-C	9.06 (4.8)	10.2 (5.0)	10.7 (5.1)	0.025	9.2 (4.8)	9.9 (5.0)	10.5 (5.1)	0.223	12.1 (5.1)	13.6 (5.7)	12.1 (4.5)	0.957	12.3 (5.0)	11.6 (5.0)	13.0 (5.0)	0.421
IDL-TG	11.0 (4.0)	12.0 (4.0)	11.7 (4.1)	0.210	11.0 (3.8)	11.5 (4.1)	11.9 (4.4)	0.323	13.4 (3.9)	15.0 (4.5)	13.4 (3.7)	0.957	13.5 (3.8)	13.1 (3.8)	14.3 (4.1)	0.322
Other atherogenic variables																
Non-HDL-P (nmol/L)	1213.0 (262.0)	1294.0 (249.0)	1348.0 (312.0)	0.001	1224.0 (264.0)	1292.0 (293.0)	1308.0 (287.0)	0.128	1415 (269)	1415 (252)	1379 (244)	0.862	1442.0 (254.0)	1365.0 (261.0)	1404.0 (250.0)	0.743
Total-P/HDL-P	42.7 (13.1)	42.3 (11.0)	45.6 (14.5)	0.174	42.7 (12.7)	44.7 (14.8)	42.9 (11.6)	0.775	48.7 (14.8)	47.6 (13.8)	46.1 (11.0)	0.862	48.4 (13.0)	45.9 (13.3)	47.9 (13.2)	0.956
LDL-P/HDL-P	41.2 (12.4)	41.1 (10.4)	44.5 (14.2)	0.120	41.4 (12.1)	43.3 (14.1)	41.7 (11.3)	0.775	47.0 (14.2)	45.5 (12.7)	44.6 (10.8)	0.862	46.8 (12.5)	44.3 (12.6)	46.1 (12.6)	0.950

Data are shown as mean (SD). HDL high-density lipoprotein, HDL-C cholesterol content in HDL, HDL-P HDL particles, HDL-TG triglyceride content in HDL, IDL intermediate-density lipopro-tein, IDL-C cholesterol content in IDL, IDL-TG triglyceride content in IDL, LDL low-density lipo-protein, LDL-C cholesterol content in LDL, LDL-P LDL particles, LDL-TG triglyceride content in LDL, NMR nuclear magnetic resonance, VLDL very lowdensity lipoprotein, VLDL-C cholesterol content in VLDL, VLDL-P VLDL particles, VLDL-TG triglyceride content in VLD. T1=poor adherence, T2=moderate adherence, and T3=high adherance

Table S7: The advanced lipoprotein profile, the alternate Mediterranean Diet score and the alternate Healthy Eating Index by age groups in men

	aMED								aHEI							
	<50 years				≥50 years				<50 years				≥50 years			
**Characteristics**	**T1 [0-4] (N= 173)**	**T2 [4] (N= 55)**	**T3 [5-9] (N=90)**	**P**	**T1 [0-4] (N = 118)**	**T2 [4] (N=67)**	**T3 [5-9] (N=128)**	**p**	**T1 [20-38] (N= 140)**	**T2 [38-44] (N= 109)**	**T3 [44-68] (N=69)**	**P**	**T1 [20-38] (N = 66)**	**T2 [38-44] (N=94)**	**T3 [44-68] (N=153)**	**p**
VLDL-P number (nmol/L)																
Total	54.1 (50.5)	52.5 (51.5)	46.5 (31.8)	0.661	63.6 (55.6)	57.1 (39.1)	57.1 (37.7)	0.843	52.9 (51.3)	48.8 (42.5)	55.3 (42.2)	0.970	61.4 (54.3)	62.0 (45.9)	56.1 (35.2)	0.901
Large	1.3 (1.1)	1.3 (1.1)	1.1 (0.7)	0.661	1.5 (1.2)	1.4 (0.8)	1.4 (0.8)	0.843	1.3 (1.2)	1.2 (0.9)	1.3 (0.9)	0.970	1.5 (1.1)	1.5 (0.9)	1.3 (0.7)	0.901
Medium	5.7 (7.3)	5.5 (8.8)	4.7 (3.5)	0.661	6.3 (9.9)	5.5 (5.0)	5.5 (4.1)	0.843	5.5 (7.7)	5.3 (7.1)	5.3 (4.0)	0.970	6.4 (10.6)	5.4 (4.2)	5.5 (3.5)	0.901
Small	47.1 (42.7)	45.7 (43.0)	40.6 (28.3)	0.661	55.8 (46.0)	50.2 (34.0)	50.3 (33.4)	0.843	46.1 (43.2)	42.4 (35.3)	48.7 (38.2)	0.970	53.5 (43.7)	55.1 (41.3)	49.3 (31.4)	0.901
VLDL-P composition (mg/dL)																
VLDL-C	13.2 (13.5)	13.1 (14.0)	11.3 (10.2)	0.661	15.9 (15.5)	13.6 (10.0)	14.7 (12.2)	0.895	12.8 (13.5)	12.1 (12.0)	13.5 (12.8)	0.970	15.2 (14.7)	15.2 (13.6)	14.3 (10.9)	0.951
VLDL-TG	79.0 (78.6)	76.3 (81.6)	67.0 (44.0)	0.661	92.1 (89.0)	82.5 (58.6)	81.4 (52.2)	0.843	77.2 (80.6)	71.1 (66.9)	79.4 (57.7)	0.970	89.6 (90.2)	88.4 (63.3)	80.1 (49.0)	0.901
VLDL-P size (nm)	42.1 (0.2)	42.2 (0.3)	42.1 (0.3)	0.905	42.1 (0.2)	42.1 (0.2)	42.1 (0.2)	0.993	42.1 (0.2)	42.2 (0.2)	42.1 (0.2)	0.970	42.1 (0.2)	42.0 (0.2)	42.1 (0.2)	0.951
LDL-P number (nmol/L)																
Total	1325.0 (251.0)	1295.0 (286.0)	1369.0 (300.0)	0.661	1322.0 (215.0)	1312.0 (278.0)	1281.0 (246.0)	0.843	1324.0 (271.0)	1338.0 (249.0)	1329.0 (321.0)	0.970	1336.0 (231.0)	1258.0 (247.0)	1311.0 (246.0)	0.901
Large	181.0 (32.8)	179.0 (37.3)	186.0 (38.8)	0.661	180.0 (30.4)	174.0 (35.7)	174.0 (32.7)	0.843	181.0 (33.8)	183.0 (32.7)	180.0 (43.9)	0.970	180.0 (32.3)	168.0 (30.1)	180.0 (33.8)	0.951
Medium	399.0 (127.0)	388.0 (140.0)	429.0 (149.0)	0.661	393.0 (122.0)	387.0 (147.0)	362.0 (114.0)	0.843	401.0 (131.0)	410.0 (125.0)	401.0 (169.0)	0.970	396.0 (125.0)	357.0 (121.0)	384.0 (130.0)	0.901
Small	745.0 (139.0)	728.0 (145.0)	754.0 (148.0)	0.905	749.0 (110.0)	751.0 (134.0)	746.0 (138.0)	0.993	742.0 (150.0)	744.0 (129.0)	748.0 (148.0)	0.970	760.0 (123.0)	733.0 (131.0)	747.0 (124.0)	0.901
LDL-P composition (mg/dL)																
LDL-C	128.0 (24.6)	126.0 (28.4)	133.0 (29.9)	0.661	126.0 (22.9)	125.0 (28.6)	121.0 (23.9)	0.843	128.0 (25.8)	130.0 (24.9)	128.0 (33.1)	0.970	128.0 (24.0)	118.0 (25.4)	125.0 (24.4)	0.901
LDL-TG	15.8 (4.8)	15.3 (5.2)	16.5 (5.3)	0.661	16.9 (4.4)	16.6 (4.8)	16.3 (5.0)	0.843	15.8 (5.2)	15.8 (4.6)	16.2 (5.7)	0.970	16.7 (4.5)	16.3 (4.5)	16.9 (5.1)	0.951
LDL-P size (nm)	20.9 (0.3)	20.9 (0.3)	20.9 (0.3)	0.717	20.9 (0.3)	20.8 (0.3)	20.8 (0.2)	0.843	20.9 (0.3)	20.9 (0.3)	20.9 (0.3)	0.970	20.9 (0.3)	20.8 (0.3)	20.9 (0.3)	0.997
HDL-P number (µmol/L)																

Total	27.2 (5.3)	26.8 (5.3)	28.5 (5.4)	0.661	27.0 (5.7)	26.9 (5.0)	27.2 (5.2)	0.993	27.5 (5.2)	27.2 (5.1)	27.7 (6.1)	0.970	27.9 (5.9)	26.1 (4.6)	26.9 (5.1)	0.901
Large	0.3 (0.1)	0.3 (0.0)	0.3 (0.1)	0.717	0.3 (0.0)	0.3 (0.0)	0.3 (0.0)	0.993	0.3 (0.1)	0.3 (0.0)	0.3 (0.1)	0.970	0.3 (0.0)	0.3 (0.0)	0.3 (0.0)	0.901
Medium	8.4 (1.7)	8.3 (1.8)	8.7 (1.9)	0.661	8.6 (2.0)	8.4 (1.8)	8.6 (2.0)	0.998	8.5 (1.8)	8.4 (1.7)	8.5 (2.03)	0.970	8.6 (2.0)	8.4 (1.9)	8.6 (2.0)	0.963
Small	18.6 (4.2)	18.2 (4.2)	19.5 (4.1)	0.661	18.2 (4.4)	18.2 (4.0)	18.4 (4.0)	0.993	18.7 (4.2)	18.6 (4.00	19.0 (4.50	0.970	19.1 (4.6)	17.4 (3.7)	18.0 (3.7)	0.901
HDL-P composition (mg/dL)																
HDL-C	51.9 (11.0)	51.5 (11.2)	55.1 (12.3)	0.661	51.0 (12.6)	51.0 (10.8)	51.3 (11.6)	0.993	52.7 (10.7)	52.5 (11.1)	52.7 (13.9)	0.970	52.8 (12.7)	48.7 (10.1)	51.2 (11.8)	0.901
HDL-TG	12.3 (4.9)	11.8 (4.6)	12.2 (4.4)	0.905	13.4 (4.6)	12.8 (3.4)	13.4 (4.8)	0.993	12.3 (4.83	11.6 (4.5)	12.8 (4.9)	0.970	13.2 (4.3)	13.7 (5.5)	12.9 (3.4)	0.951
HDL-P size (nm)	8.2 (0.1)	8.2 (0.1)	8.2 (0.1)	0.905	8.2 (0.1)	8.2 (0.1)	8.2 (0.1)	0.993	8.2 (0.1)	8.2 (0.1)	8.2 (0.1)	0.970	8.2 (0.1)	8.2 (0.1)	8.2 (0.1)	0.901
IDL-P composition (mg/dL)																
IDL-C	9.6 (4.2)	9.5 (4.3)	10.0 (4.5)	0.840	11.6 (4.6)	11.7 (3.8)	11.4 (4.6)	0.993	9.7 (4.2)	9.4 (4.0)	10.1 (5.0)	0.970	11.7 (4.6)	11.4 (4.3)	11.6 (4.3)	0.951
IDL-TG	11.3 (3.4)	10.9 (3.5)	11.5 (3.7)	0.905	13.0 (3.8)	12.8 (3.4)	13.0 (3.9)	0.998	11.3 (3.4)	10.9 (3.2)	11.9 (4.3)	0.970	12.9 (3.6)	12.9 (4.1)	13.1 (3.7)	0.951
Other atherogenic variables																
Non-HDL-P (nmol/L)	1351.0 (255.0)	1320.0 (290.0)	1387.0 (309.0)	0.717	1359.0 (215.0)	1342.0 (276.0)	1311.0 (255.0)	0.843	1349.0 (275.0)	1359.0 (255.0)	1357.0 (324.0)	0.970	1369.0 (238.0)	1294.0 (239.0)	1340.0 (254.0)	0.901
Total-P/HDL-P	52.2 (12.8)	53.0 (17.8)	51.7 (15.9)	0.905	53.2 (12.5)	52.4 (13.4)	51.4 (15.7)	0.843	51.7 (13.7)	53.0 (14.8)	52.3 (17.1)	0.970	52.2 (13.9)	52.1 (12.5)	52.9 (15.0)	0.951
LDL-P/HDL-P	50.2 (12.4)	50.8 (16.9)	49.9 (15.2)	0.953	50.7 (12.0)	50.1 (12.7)	49.2 (14.9)	0.843	49.7 (13.3)	51.0 (14.0)	50.1 (16.3)	0.970	49.9 (13.2)	49.6 (12.2)	50.6 (14.2)	0.951

Data are shown as mean (SD). HDL high-density lipoprotein, HDL-C cholesterol content in HDL, HDL-P HDL particles, HDL-TG triglyceride content in HDL, IDL intermediate-density lipopro-tein, IDL-C cholesterol content in IDL, IDL-TG triglyceride content in IDL, LDL low-density lipo-protein, LDL-C cholesterol content in LDL, LDL-P LDL particles, LDL-TG triglyceride content in LDL, NMR nuclear magnetic resonance, VLDL very lowdensity lipoprotein, VLDL-C cholesterol content in VLDL, VLDL-P VLDL particles, VLDL-TG triglyceride content in VLD. T1=poor adherence, T2=moderate adherence, and T3=high adherance

Table S8: The advanced lipoprotein profile, the alternate Mediterranean Diet score and the alternate Healthy Eating Index by age groups in women

	aMED								aHEI							
	<50 years				≥50 years				<50 years				≥50 years			
**Characteristics**	**T1 [0-4] (N= 173)**	**T2 [4] (N= 55)**	**T3 [5-9] (N=90)**	**P**	**T1 [0-4] (N = 118)**	**T2 [4] (N=67)**	**T3 [5-9] (N=128)**	**p**	**T1 [20-38] (N= 140)**	**T2 [38-44] (N= 109)**	**T3 [44-68] (N=69)**	**P**	**T1 [20-38] (N = 66)**	**T2 [38-44] (N=94)**	**T3 [44-68] (N=153)**	**p**
VLDL-P number (nmol/L)																
Total	39.4 (27.2)	36.4 (20.2)	35.0 (23.5)	0.210	47.7 (25.2)	60.8 (42.9)	44.4 (27.5)	0.868	36.6 (23.4)	38.7 (27.2)	38.1 (25.2)	0.775	45.1 (26.1)	46.0 (28.2)	52.9 (34.5)	0.322
Large	1.0 (0.6)	0.9 (0.4)	0.9 (0.5)	0.086	1.1 (0.5)	1.4 (0.9)	1.1 (0.6)	0.868	0.9 (0.5)	1.0 (0.6)	1.0 (0.5)	0.775	1.1 (0.5)	1.1 (0.6)	1.25 (0.7)	0.322
Medium	3.9 (2.8)	3.6 (2.0)	3.3 (2.2)	0.072	4.4 (2.6)	5.5 (4.4)	4.4 (3.0)	0.957	3.8 (2.4)	3.7 (2.7)	3.6 (2.4)	0.775	4.2 (2.4)	4.6 (3.4)	4.9 (3.4)	0.378
Small	34.5 (24.3)	31.9 (18.2)	30.9 (21.1)	0.236	42.2 (22.7)	53.9 (38.2)	38.9 (24.3)	0.862	31.9 (20.9)	34.1 (24.3)	33.5 (22.6)	0.775	39.8 (23.7)	40.3 (24.7)	46.8 (30.9)	0.322
VLDL-P composition (mg/dL)																
VLDL-C	9.6 (8.8)	8.6 (7.2)	8.6 (7.2)	0.357	11.9 (8.3)	15.1 (12.2)	10.6 (8.7)	0.862	8.9 (8.0)	9.2 (8.4)	9.3 (8.0)	0.775	11.0 (8.4)	11.0 (8.4)	13.1 (10.6)	0.325
VLDL-TG	56.8 (37.5)	52.1 (26.9)	49.4 (32.3)	0.164	67.6 (34.1)	86.5 (60.8)	64.0 (38.5)	0.868	52.7 (32.0)	55.3 (37.7)	54.1 (34.5)	0.775	64.0 (35.1)	66.3 (40.9)	75.2 (47.9)	0.322
VLDL-P size (nm)	42.1 (0.2)	42.1 (0.2)	42.1 (0.2)	0.027	42.1 (0.2)	42.0 (0.2)	42.1 (0.2)	0.862	42.2 (0.2)	42.1 (0.2)	42.1 (0.2)	0.085	42.1 (0.2)	42.1 (0.2)	42.1 (0.2)	0.473
LDL-P number (nmol/L)																
Total	1204.0 (252.0)	1290.0 (241.0)	1344.0 (304.0)	<0.001	1398.0 (260.0)	1386.0 (243.0)	1366.0 (245.0)	0.862	1218.0 (255.0)	1284.0 (284.0)	1302.0 (277.0)	0.128	1428.0 (249.0)	1351.0 (253.0)	1382.0 (247.0)	0.583
Large	178.0 (31.9)	189.0 (35.7)	195.0 (37.9)	0.001	195.0 (36.2)	187.0 (37.1)	190.0 (34.6)	0.862	182.0 (33.6)	185.0 (36.7)	189.0 (35.4)	0.323	201.0 (38.1)	186.0 (34.7)	190.0 (34.8)	0.325
Medium	386.0 (124.0)	438.0 (123.0)	470.0 (155.0)	<0.001	466.0 (135.0)	445.0 (132.0)	457.0 (132.0)	0.873	405.0 (131.0)	426.0 (150.0)	437.0 (133.0)	0.303	492.0 (141.0)	436.0 (125.0)	456.0 (132.0)	0.384
Small	640.0 (131.0)	663.0 (112.0)	679.0 (141.0)	0.050	737.0 (131.0)	754 (123)	720.0 (117.0)	0.862	631.0 (122.0)	673.0 (132.0)	676.0 (141.0)	0.107	735.0 (109.0)	728.0 (134.0)	736.0 (125.0)	0.907
LDL-P composition (mg/dL)																
LDL-C	119.0 (24.8)	128.0 (24.1)	133.0 (30.6)	<0.001	136.0 (26.1)	133.0 (25.0)	133.0 (25.7)	0.862	121.0 (25.5)	126.0 (29.0)	129.0 (26.9)	0.155	141.0 (25.8)	131.0 (25.1)	134.0 (25.7)	0.378
LDL-TG	15.3 (5.0)	17.1 (5.1)	17.9 (5.4)	<0.001	18.5 (5.1)	18.8 (5.1)	18.4 (4.3)	0.957	15.9 (5.3)	16.6 (5.3)	16.9 (5.1)	0.323	19.0 (5.4)	17.6 (4.8)	18.9 (4.4)	0.834
LDL-P size (nm)	21.1 (0.3)	21.1 (0.2)	21.2 (0.2)	0.028	21.0 (0.3)	21.0 (0.3)	21.0 (0.3)	0.957	21.1 (0.2)	21.1 (0.2)	21.1 (0.2)	0.562	21.1 (0.3)	21.0 (0.25)	21.0 (0.26)	0.325
HDL-P number (µmol/L)																
Total	30.4 (6.0)	32.6 (6.7)	31.2 (5.5)	0.231	31.1 (6.2)	31.5 (5.3)	31.4 (5.0)	0.905	30.6 (6.3)	30.9 (6.0)	32.0 (5.3)	0.323	31.5 (5.5)	31.6 (6.0)	31.1 (5.3)	0.724

Large	0.3 (0.1)	0.3 (0.1)	0.3 (0.1)	0.007	0.30 (0.1)	0.3 (0.0)	0.3 (0.0)	0.868	0.3 (0.1)	0.3 (0.1)	0.3 (0.1)	0.128	0.3 (0.1)	0.3 (0.1)	0.3 (0.0)	0.907
Medium	10.3 (2.3)	11.0 (2.9)	10.7 (2.2)	0.177	10.1 (2.5)	9.71 (2.0)	10.1 (2.1)	0.957	10.3 (2.4)	10.5 (2.4)	10.8 (2.2)	0.323	10.1 (2.1)	10.2 (2.6)	9.87 (2.1)	0.583
Small	19.9 (4.3)	21.3 (4.3)	20.2 (3.8)	0.357	20.7 (4.3)	21.5 (3.9)	21.1 (3.8)	0.868	20.0 (4.4)	20.1 (4.1)	20.9 (3.8)	0.323	21.2 (4.1)	21.1 (4.2)	20.9 (4.0)	0.834
HDL-P composition (mg/dL)																
HDL-C	59.7 (13.4)	64.4 (15.8)	61.9 (11.6)	0.177	60.5 (14.7)	59.1 (12.8)	60.7 (11.8)	0.957	60.3 (13.8)	61.0 (14.2)	63.0 (11.2)	0.323	61.0 (12.8)	61.7 (14.8)	59.2 (12.2)	0.459
HDL-TG	14.4 (4.6)	15.1 (4.2)	14.6 (4.9)	0.645	14.7 (4.0)	16.0 (3.4)	14.9 (3.8)	0.873	14.3 (4.7)	14.5 (4.3)	15.2 (5.0)	0.337	14.9 (4.2)	14.5 (3.1)	15.4 (4.0)	0.444
HDL-P size (nm)	8.2 (0.1)	8.2 (0.1)	8.3 (0.1)	0.563	8.2 (0.1)	8.2 (0.1)	8.2 (0.1)	0.868	8.2 (0.1)	8.2 (0.1)	8.2 (0.1)	0.855	8.2 (0.1)	8.2 (0.1)	8.2 (0.1)	0.834
IDL-P composition (mg/dL)																
IDL-C	9.06 (4.8)	10.2 (5.0)	10.7 (5.1)	0.025	12.1 (5.1)	13.6 (5.7)	12.1 (4.5)	0.957	9.2 (4.8)	9.9 (5.0)	10.5 (5.1)	0.223	12.3 (5.0)	11.6 (5.0)	13.0 (5.0)	0.421
IDL-TG	11.0 (4.0)	12.0 (4.0)	11.7 (4.1)	0.210	13.4 (3.9)	15.0 (4.5)	13.4 (3.7)	0.957	11.0 (3.8)	11.5 (4.1)	11.9 (4.4)	0.323	13.5 (3.8)	13.1 (3.8)	14.3 (4.1)	0.322
Other atherogenic variables																
Non-HDL-P (nmol/L)	1213.0 (262.0)	1294.0 (249.0)	1348.0 (312.0)	0.001	1415 (269)	1415 (252)	1379 (244)	0.862	1224.0 (264.0)	1292.0 (293.0)	1308.0 (287.0)	0.128	1442.0 (254.0)	1365.0 (261.0)	1404.0 (250.0)	0.743
Total-P/HDL-P	42.7 (13.1)	42.3 (11.0)	45.6 (14.5)	0.174	48.7 (14.8)	47.6 (13.8)	46.1 (11.0)	0.862	42.7 (12.7)	44.7 (14.8)	42.9 (11.6)	0.775	48.4 (13.0)	45.9 (13.3)	47.9 (13.2)	0.956
LDL-P/HDL-P	41.2 (12.4)	41.1 (10.4)	44.5 (14.2)	0.120	47.0 (14.2)	45.5 (12.7)	44.6 (10.8)	0.862	41.4 (12.1)	43.3 (14.1)	41.7 (11.3)	0.775	46.8 (12.5)	44.3 (12.6)	46.1 (12.6)	0.950

Data are shown as mean (SD). HDL high-density lipoprotein, HDL-C cholesterol content in HDL, HDL-P HDL particles, HDL-TG triglyceride content in HDL, IDL intermediate-density lipopro-tein, IDL-C cholesterol content in IDL, IDL-TG triglyceride content in IDL, LDL low-density lipo-protein, LDL-C cholesterol content in LDL, LDL-P LDL particles, LDL-TG triglyceride content in LDL, NMR nuclear magnetic resonance, VLDL very lowdensity lipoprotein, VLDL-C cholesterol content in VLDL, VLDL-P VLDL particles, VLDL-TG triglyceride content in VLD. T1=poor adherence, T2=moderate adherence, and T3=high adherance
